# A comparison of impact and risk assessment methods based on the IMO Guidelines and EU invasive alien species risk assessment frameworks

**DOI:** 10.7717/peerj.6965

**Published:** 2019-06-10

**Authors:** Greta Srėbalienė, Sergej Olenin, Dan Minchin, Aleksas Narščius

**Affiliations:** 1 Marine Research Institute, Klaipėda University, Klaipėda, Lithuania; 2 Marine Organism Investigations, Marina Village, Ballina, Killaloe, Co Clare, Ireland

**Keywords:** Risk assessment, Invasive alien species, Impact, IMO guidelines, Policy relevance, Biosecurity, EU regulation

## Abstract

A comparative analysis of two risk assessment (RA) frameworks developed to support the implementation of the international Ballast Water Management Convention (BWMC) and European Regulation on Invasive Alien Species (IAS) was performed. This analysis revealed both differences and similarities between the IMO Risk Assessment Guidelines (IMO, 2007) and EU Regulation supplement on RA of IAS (EU, 2018) in RA approaches, key principles, RA components and categories of IAS impacts recommended for assessment. The results of this analysis were used to produce a common procedure for the evaluation of the bioinvasion risk and impact assessment methods intended to support international, regional and/or national policy on IAS. The procedure includes a scoring scheme to assess compliance with the key principles, RA components and categories of bioinvasion impacts taken into account by the methods. In these methods the categories of impacts on human health and economy are underrepresented comparing with impacts on environment.

## Introduction

There is a need for standardized methods to measure the magnitude of invasive species impacts and to assess their risk. This has promoted a new direction in applied invasion ecology. More than seventy tools have been developed during recent decades aimed at bioinvasion impact and risk assessment (Roy et al., 2017). They are named variously as “protocols” (Verbrugge et al., 2012), “frameworks” (Dahlstrom, Hewitt & Campbell, 2011), “tool” (e.g., Drolet et al., 2016), “kit” (e.g., Copp et al., 2009), “scheme” (e.g., Baker et al., 2008), “system” (e.g., Nentwig, Kühnel & Bacher, 2010), “index” (Olenin, Minchin & Daunys, 2007), etc. In this account, we have termed these all as “bioinvasion risk and impact assessment methods”, or “the methods” which may differ according to the geographical scale from local to regional and global, and by realm, either terrestrial or aquatic, or both. The principal aim of these methods was to provide information to support management decisions by prioritizing invasive species, choosing prevention measures, compiling target lists and assessing their overall environmental status (Olenin, Minchin & Daunys, 2007; Molnar et al., 2008).

While the number of bioinvasion risk and impact assessment methods increases, there are difficulties in choosing the most appropriate method that best corresponds to the basic principles of risk assessment (RA). Some international legislation and administrative documents provide guidelines and methodologies for measuring bioinvasion risk and impact assessment methods (Dahlstrom, Hewitt & Campbell, 2011; Verbrugge et al., 2012; Tollington et al., 2017). For example, the International Maritime Organization (IMO) adopted the International Convention for the Control and Management of Ships’ Ballast Water and Sediments (BWMC) aimed at reducing the spread of harmful aquatic organisms and pathogens (HAOPs) (IMO, 2004). Later the IMO developed Guidelines for risk assessment outlining methods enabling managers to identify risk scenarios and make decisions on granting ballast water management exemptions under BWMC Regulation A-4 (G7) (IMO, 2007), which came into force in September 2017 (IMO, 2017). Similarly, at the European level, the EU Parliament adopted the Regulation on the prevention and management of the introduction and spread of invasive alien species (IAS) (EU, 2014), and a few years later, provided a supplementary document with regard to IAS risk assessment (European Union, 2018).

Both the IMO Guidelines (IMO, 2007) and EU Regulation (European Union, 2018) provide a framework for RA, indicating RA principles, data needs, RA elements and the scope of each assessment. While the IMO Guidelines (IMO, 2007) is vector-specific, and are focused on minimizing the risk of HAOPs transfered in ballast water, the EU Regulation (European Union, 2018) is more generic, including all possible habitats (marine, freshwater, terrestrial) and a complete array of possible vectors for all taxa, with the aim of harmonizing common risk assessment methods.

Barry et al. (2008) reviewed eight “ballast water risk assessment systems” developed from 1992 to 2004. Later, David & Gollasch (2015) completed a review including four additional methods and assessed their compliance with the BWMC requirements. However, since then, new methods for ballast water RA (e.g., Drolet et al., 2016; Verna et al., 2016; Simard et al., 2017) and the updates of earlier reviewed methods have appeared (e.g., David & Gollasch, 2015). Dahlstrom, Hewitt & Campbell (2011) took a more general approach assessing the “biosecurity risk assessment frameworks” based on fourteen international, regional and national legal instruments. They proposed a set of recommendations to develop aquatic biosecurity risk frameworks in accord with mandates established by international bodies. With the advent of the EU Regulation (European Union, 2018), there is a need for an approach that enables comparison of the different bioinvasion risk and impact assessment methods, which ensures compliance with legislative and administrative requirements.

This paper aims to develop a general framework for evaluating and comparing bioinvasion risk and impact assessment methods. We first analyzed the IMO Guidelines (IMO, 2007) and the EU Regulation (European Union, 2018) by comparing (i) the key principles of RA, (ii) assessment components, (iii) and categories of bioinvasion impacts. In this context, the assessment components are “data necessary to enable a RA” (IMO, 2007) or “the common elements that are to be considered in the risk assessment” (European Union, 2018), such as reproduction and spread, pathways, distribution, etc.

To our knowledge, this is the first comparison of these two frameworks, based on the legal instruments (IMO, 2007 and European Union, 2018, respectively) intended to minimize bioinvasion risk. In this account we develop a common procedure that amalgamates the elements of both RA frameworks. The procedure includes a scoring scheme to assess how the methods comply with (i) the key principles, (ii) in which extent they cover the RA components and (iii) what categories of bioinvasion impacts they take into account.

## Methods

### Setting the scene: comparison of the IMO and EU risk assessment frameworks

**Figure 1:**
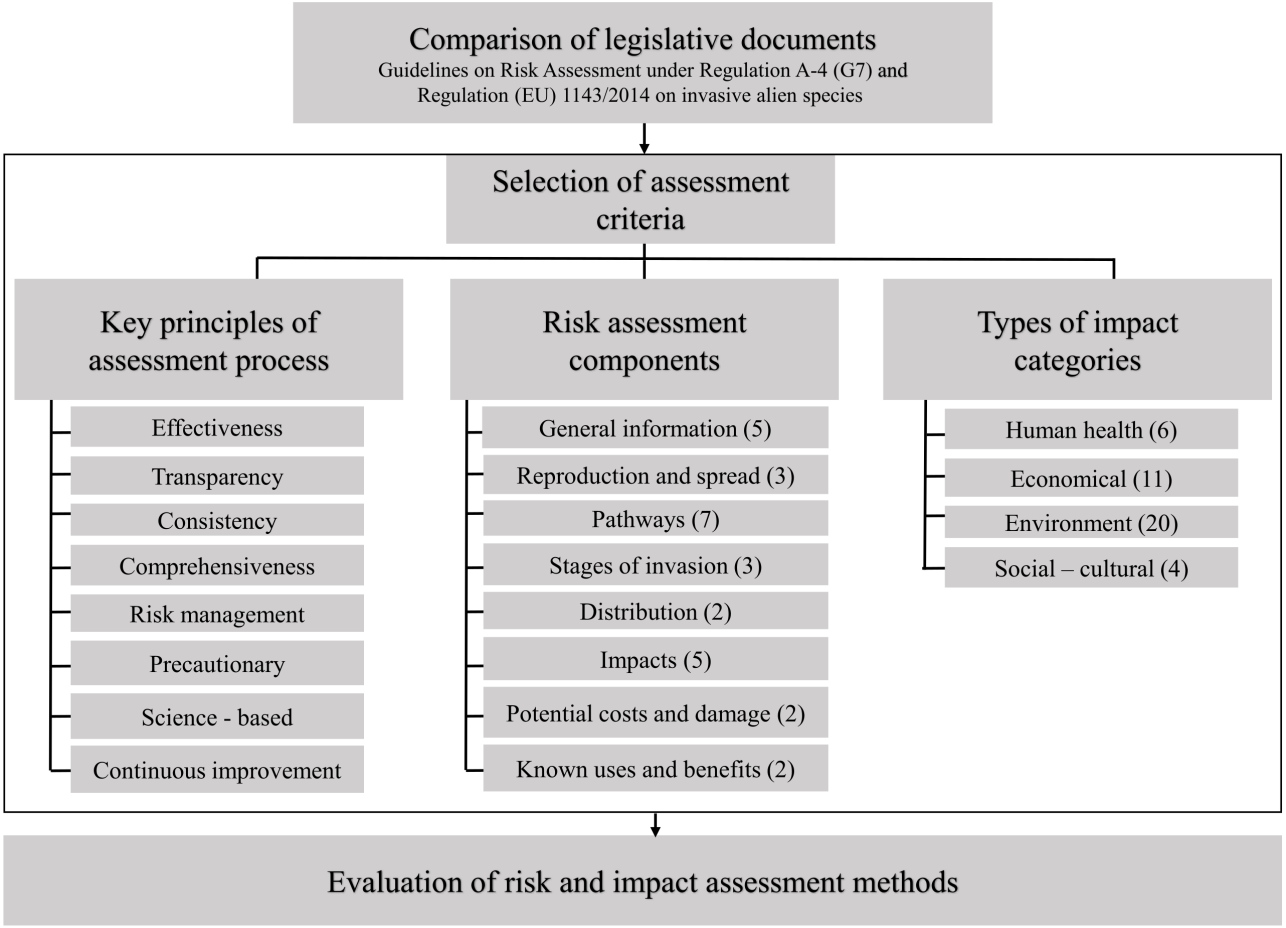
A stepwise process of the evaluation of bioinvasion risk and impact assessment methods: comparison of legislative documents, selection of criteria and evaluation. The number of elements in risk assessment components and categories in types of impact is given in brackets (listed in Tables S1 and S2, accordingly).

The frameworks of both the IMO (2007) and European Union (2018) regulations were compared to provide a support for a common evaluation procedure (Fig. 1; see also Tables S1 and  S2). Accordingly, we screened the RA frameworks as follows: (i) key principles of an assessment process, (ii) assessment components and, (iii) categories of bioinvasion impacts to be taken into account using the above RA frameworks.

### The evaluation procedure

Based on the comparison of the IMO Guidelines and the EU Regulation, the evaluation procedure included eight key principles of the IMO Guidelines, twenty-nine RA components of the EU Regulation and four main bioinvasion impact types, compiled from both documents (Fig. 1). In addition, we incorporated impact categories as proposed in earlier risk assessment frameworks (Emerton & Howard, 2008; David & Gollasch, 2015; Olenin et al., 2016; Vilà & Hulme, 2017). In all, 41 categories were defined: human health (six categories), economy (11), environment (20), social-cultural aspects (four). Descriptions of the impact categories are provided in the supplementary table (Table S2).

We developed a scoring scheme in order to assess the compliance with each of the eight key principles (Table 1). The RA components and the categories of impact were considered to be either present or absent based on the original description of the selected. The overall ranking of selected methods is based on an accumulated score, and expressed as a percentage of compliance with our selected criteria. We discuss the advantages and limitations of this approach.

**Table 1:** A scoring system to assess the compliance to the key principles of the risk assessment. “1” the method fully meets a criterion, “0” the method is not compliant with criteria.

Key principle	Definition by IMO (2007)	Scoring criteria	
Effectiveness	*That risk assessments accurately measure the risks to the extent necessary to achieve an appropriate level of protection.*	1	definitions of all parameters provided, the calculation scheme is clear, the result is obtained either automatically using an online platform or by a questionnaire.
		0	definitions of all parameters are not provided, no calculation included, overall result is not obtained.
Transparency	*That the reasoning and evidence supporting the action recommended by risk assessments, and areas of uncertainty (and their possible consequences to those recommendations), are clearly documented and made available to decision-makers.*	1	the reasoning and evidence supporting the assessment is documented and (or) is available via a free online information system or on request from the authors.
		0	not compliant.
Consistency	*That risk assessments achieve a uniform high level of performance, using a common process and methodology.*	1	the consistency of a method was tested by assessing the repeatability of the test outcome, the results are published in peer-reviewed literature.
		0	the assessment of the consistency of a method is not available publically.
Comprehensiveness	*That the full range of values, including economic, environmental, social and cultural, are considered when assessing risks and making recommendations.*	1	the method considers all four categories of risks and impacts (human health, economic, environmental /ecological, social and cultural aspects).
		0	a method considers less than four categories.
Risk management	*That low risk scenarios may exist, but zero risk is not obtainable, and as such risk should be managed by determining the acceptable level of risk in each instance.*	1	the method clearly defines the level of risk /bioinvasion impact that can be used for the risk management.
		0	no definition of the magnitude of risk /bioinvasion impact is given.
Precautionary	*That risk assessments incorporate a level of precaution when making assumptions, and making recommendations, to account for uncertainty, unreliability, and inadequacy of information. The absence of, or uncertainty in, any information should therefore be considered an indicator of potential risk.*	1	incorporates level of confidence for all risk assessment steps, including the level of confidence for the final risk score, clear instructions how to define uncertainty.
		0	no level of confidence is taken into account.
Science-based	*That risk assessments are based on the best available information that has been collected and analyzed using scientific methods.*	1	at least part of the assessment requires quantitative experimental and/or field study data, or the review of scientific literature.
		0	the method takes into account impacts and risks of invasive species based only on expert judgement, no quantitative experimental and/or field studies data used.
Continuous improvement	*Any risk model should be periodically reviewed and updated to account for improved understanding.*	1	the method has been updated since publication of the original version.
		0	only original version exists, has no updated version until know.

### Selection and review of the bioinvasion impact and risk assessment methods

To select the bioinvasion impact and risk assessment methods for the analysis we used the list of the most relevant methods identified Roy et al. (2017) and the COST Action Alien Challenge TD1209. They performed a worldwide literature search for the methods of invasive species risk assessment (RA), and crosschecked the references for additional relevant publications to obtain twenty-nine original RA methods. We used these methods for an analysis based on the following criteria: (a) the method is applicable for the aquatic realm; (b) the assessment results are either in a quantitative or in qualitative form, and (c) it takes into account at least one of the four categories of bioinvasion impacts. From this preliminary analysis we selected nine methods out of the 29 reviewed by Roy et al. (2017) and we searched the literature to include any further methods which were not considered relevant in their review, yet met our criteria. We found fifteen methods suitable for our analysis (Table 2). The selected methods represent different regions and we recognize that there might be further methods worldwide which did not come to our attention. It should be noted that our main goal was to test the evaluation procedure on a sufficient number of methods.

We refer to each method by an acronym (Table 2), while some have changed their names with further development, for example, AS-ISK (Copp et al., 2016) was originally known as FISK “Fish Invasiveness Screening Kit” (Copp et al., 2009) and the Biopollution level (BPL) (Olenin, Minchin & Daunys, 2007) was later computerized and renamed as the Bioinvasion impact/ Biopollution assessment system, BINPAS (Narščius et al., 2012). Most of the methods (75%) were published in peer-reviewed journals, one as a book chapter, and three appeared in national or international environmental reports. The methods were divided into three groups, according to their assessment goals as: (1) the screening tools (AS-ISK, CMIST, HARMONIA+), (2) risk assessment tools (GB NNRA, TRAAIS, SBRA, WISC, RABW), (3) impact assessment indexes/schemes (CIMPAL, BINPAS, GISS, GABLIS, GEIAA, GISS IUCN, GLOTSS).

**Table 2:** Summary of the risk and impact assessment methods.

Title of the method	Acronym	Key reference	Assessment goal	Method assessment	Example of the use
Aquatic Species Invasiveness Screening Kit	AS-ISK	Copp et al. (2016)	Screening/horizon scanning	Excel sheet	Tricarico et al. (2010), Papavlasopoulou et al. (2014)
Biological Invasion Impact/Biopollution Assessment System	BINPAS	Olenin, Minchin & Daunys (2007)	Impact assessment	Online tool	Olenina et al. (2010), Zaiko et al. (2011), Minchin & White (2014)
Cumulative impacts of invasive alien species	CIMPAL	Katsanevakis, Tempera & Teixeira (2016)	Impact assessment	Excel sheet	Katsanevakis, Tempera & Teixeira (2016)
Canadian Marine Invasive Screening Tool	CMIST	Drolet et al. (2016)	Impact assessment/ screening tool	Online tool	Drolet et al. (2016)
German–Austrian Black List Information System	GABLIS	Essl et al. (2011)	Impact assessment	Questionnaire	Nehring, Essl & Rabitsch (2013a), Nehring et al. (2013b); Rabitsch et al. (2013)
Full Risk Assessment Scheme for Non-native Species in Great Britain	GB NNRA	Baker et al. (2008)	Impact/risk assessment	Questionnaire	Baker et al. (2008), Mumford et al. (2010)
Norwegian Generic Ecological Impact Assessments of Alien species	GEIAA	Sandvik et al. (2013)	Impact assessment	Excel sheet, Statistical program R	Sandvik et al. (2013)
The generic impact scoring system	GISS	Nentwig, Kühnel & Bacher (2010)	Impact assessment	Questionnaire	Kumschick & Nentwig (2010), Vaes-Petignat & Nentwig (2014), Nentwig et al. (2016)
The generic impact scoring system including IUCN criteria	*GISS IUCN*	Blackburn et al. (2014)	Impact assessment	Questionnaire	Blackburn et al. (2014)
HARMONIA+	HARMONIA+	D’hondt et al. (2015)	Impact assessment/ screening tool	Online tool	D’hondt et al. (2015)
Global threat scoring system	GLOTSS	Molnar et al. (2008)	Impact assessment	Questionnaire	Molnar et al. (2008)
Risk assessment for exemptions from ballast water management	RABW	David, Gollasch & Leppäkoski (2013)	Risk assessment	Questionnaire	David, Gollasch & Leppäkoski (2013)
Species Biofouling Risk Assessment	SBRA	Hewitt et al. (2011)	Risk assessment	Questionnaire	Hewitt et al. (2011)
Trinational Risk Assessment for Aquatic Alien Invasive Species	TRAAIS	Mendoza Alfaro (2009)	Risk assessment	Questionnaire	Mendoza Alfaro (2009)
Invasive Species Impact and Prevention/Early Action Assessment Tool	WISC	WISC (2009)	Risk assessment	Questionnaire	WISC (2009)

## Results

### Similarities and differences between the two legislative documents

The screening revealed differences and similarities between two documents, which are summarized in Table 3.

**Table 3:** The analysis of the IMO guidelines and EU regulation risk assessment frameworks. The EU regulation* (italic) and IMO guidelines* (plain text) risk assessment frameworks. IC: Incorporation of the criteria; ○: criteria only in IMO Guidelines (specifically, point G7); ●: criteria only in EU regulation (specifically Article 5.1); ◗◯ criteria in both documents. IA: IMO RA approach type; ■ environmental matching risk assessment; ▴ species biogeographical risk assessment; □ species-specific risk assessment.

Assessment criteria	Comparison of criteria by IMO and EU regulation risk assessment frameworks	IC	IA
Key principles of the assessment process**	Effectiveness	○	
	*Reliable scientific information supported by references to peer reviewed scientific publications*/transparency	◗◯	
	Consistency	○	
	Comprehensiveness	○	
	Risk management	○	
	*Level of uncertainty or confidence, quality control, overall risk /* precautionary	◗◯	
	*Scientific robustness, efficiency of knowledge /* science based	◗◯	
	Continuous improvement	○	
Risk assessment components	*Species taxonomic identity, history, natural and potential range (Art 5(1) (a))*		
	1. *The description of the species*	●	
	2. *The scope of the risk assessment*	●	
	3. *Taxonomic identity of the species*	●	
	4. *Invasion history of the species, including information on countries invaded, an indication of the timeline of the first observations, establishment and spread/* information on life history and physiological tolerances, estimate potential to survive or complete its life cycle, individual species characteristics, biogeographical distributions of nonindigenous species, native species with wide biogeographical or habitat distributions, invaders in other biogeographic regions, environmental matching degree of similarity between the locations.	◗◯	■▴□
	5. *Natural and potential range of the species, an indication of the continent or part of a continent, climatic zone and habitat where the species is naturally occurring/* identify species that are present in the donor port but not in the recipient port, current distribution within biogeographic region and in other biogeographic regions, environmental conditions of the source region should be considered.	◗◯	■□
	*Reproduction, spread patterns, dynamics, an assessment of environmental conditions for reproduction and spread (Art 5(1) (b))*		
	1. *Reproduction and spread patterns: species life history and behavioral traits, ability to establish and spread, reproduction or growth strategy, dispersal capacity, longevity, environmental and climatic requirements, specialist or generalist characteristics*/information on life history and physiological limits, estimate its potential to survive or complete its life cycle, degree of similarity between the locations, the likelihood of survival and the establishment.	◗◯	■□
	2. *Reproduction patterns and following elements: suitable environmental conditions for the species reproduction exist in the risk assessment area, e.g., number of gametes, seeds, eggs or propagules, number of reproductive cycles per year*/information on life history and physiological limits, estimate its potential to survive, complete its life cycle, degree of similarity between the locations provides an indication of the likelihood of survival and establishment, compare environmental conditions to determine the likelihood ability to survive.	◗◯	■□
	3. *Spread patterns and dynamics and following elements*/information on life history and physiological tolerances to define a species physiological limits, estimate its potential to survive, complete its life cycle, degree of similarity between the locations provides an indication of the likelihood of survival and establishment, analysis of environmental conditions be followed that can tolerate extreme environmental differences.	◗◯	■□
	*Potential pathways of introduction, spread, intentional and unintentional, the associated commodities(Art 5(1) (c))*		
	1. *Relevant pathways for introduction and spread. The classification of pathways by the Convention on Biological Diversity*/identify the species that have the ability to invade and become harmful and relationship with ballast water as a vector, records of native or non-indigenous species that could be transferred through ballast water.	◗◯	▴□
	2. *Intentional pathways of introduction and following elements /* identify the species that have the ability to invade and become harmful and relationship with ballast water as a vector, records of native or non-indigenous species that could be transferred through ballast water.	◗◯	▴□
	3. *Unintentional pathways of introduction and following elements /* identify the species that have the ability to invade and become harmful and relationship with ballast water as a vector, records of native or non-indigenous species that could be transferred through ballast water.	◗◯	▴□
	4. *Commodities with which the introduction of the species is generally associated, commodities with an indication of associated risks (e.g., the volume of trade flow; the likelihood of the commodity being contaminated or acting as a vector) /* identify the species that have the ability to invade and become harmful and relationship with ballast water as a vector, seasonal variations in surface and bottom salinities, determine the full range of environmental conditions available for a potential invader.	◗◯	▴□
	5. *Intentional pathways of spread and following elements: commodities with an indication of associated risks (e.g., the volume of trade flow, the likelihood of the commodity being contaminated or acting as a vector) /* records of species that could be transferred through ballast water, the number, nature of biogeographic regions invaded, life history, physiological tolerances, physiological limits, estimate its potential to survive, complete life cycle in the recipient environment, species characteristics with the environmental conditions, determine the likelihood of transfer and survival.	◗◯	▴□
	6. *Unintentional pathways of spread and following elements /* records of species that could be transferred through ballast water in the donor biogeographic region, invaded other biogeographic regions, number and nature of biogeographic regions invaded, life history and physiological limits, estimate its potential to survive, complete its life cycle in the recipient environment.	◗◯	▴□
	7. *Commodities with which the spread of the species is generally associated, commodities with associated risks (e.g., the volume of trade; the likelihood of a commodity being contaminated or acting as vector) /* records of species that could be transferred through ballast water, life history and physiological limits, estimate its potential to survive, complete its life cycle in the recipient environment, individual species characteristics with the environmental conditions, determine the likelihood of transfer and survival.	◗◯	▴□
	*Assessment of the risk of introduction, establishment, spread in biogeographical regions in current and climate change conditions (Art 5(1) (d))*		
	1. *Assessment risks of a species introduction into, establishment, spread within relevant biogeographical regions, explanation how foreseeable climate change conditions will influence risks*/biogeographical distributions; identify potential target species in the donor regions with wide biogeographical or habitat distributions, known invaders in other biogeographic regions/ environmental conditions compared, similarity in key environmental conditions, environmental conditions for environmental matching include temperature, nutrients, oxygen or other.	◗◯	■▴□
	2. *Assessment of likely introduction, establishment and spread within a medium timeframe scenario (e.g., 30-50 years).*	◗◯	■▴□
	3. *Description of risks can be in terms of ‘likelihood’ or ‘rate’/* degree of similarity between the locations indicates the likelihood of survival and the establishment, species characteristics with the environmental conditions to determine the likelihood of transfer and survival, likelihood of target species survival, probability of viable stages entering the vessel’s ballast water tanks, probability of survival during the voyage, probability of viable stages entering the recipient port through ballast water discharge on arrival.	◗◯	□
	*Current distribution, projection of its likely future distribution (Art 5(1) (e))*		
	1. *Current distribution in the risk assessment area or in neighbouring countries*/biogeographical distributions of species that presently exist in biogeographic regions; records of invasion in biogeographic regions and ports/ biogeographic region of donor and recipient port(s); the presence of target species in the recipient port(s), port region, and biogeographic region.	◗◯	▴□
	2. *Likely future distribution in the risk assessment area or in neighbouring countries*/identify potential target species with wide biogeographical or known invaders in other biogeographic regions, the presence of target species in the recipient port(s), port region, and biogeographic region; life history information on the target species and physiological tolerances, in particular salinity and temperature, of each life stage; habitat type required by the target species and availability of habitat type in the recipient port, the likelihood of target species surviving.	◗◯	▴□
	*Adverse impact on biodiversity, ecosystem services, native species, protected sites, endangered habitats, human health, safety, economy, potential future impact (Art 5(1) (f))*		
	1. *Known impact or potential future impact on biodiversity and related ecosystem services. The potential future impact in the risk assessment area*/records of native that have the potential to affect or result in substantial ecological impacts/species of concern that may impair or damage the environment need to be identified and selected (e.g., target species). Target species should be selected for a specific port, State, or geographical region, and should be identified and agreed.	◗◯	▴□
	2. *Known impact and the assessment of the potential future impact. The magnitude of the impact scored or otherwise classified. The impact scoring or classification system include a reference to the underlying publication /* species biogeographical risk assessment compares the biogeographical distributions of nonindigenous, cryptogenic, and harmful native species that presently exist in the donor and recipient ports and biogeographic regions.	◗◯	▴
	3. *Known impact and the assessment of the potential future impact on biodiversity /* records of native species have the potential to affect, result in ecological impacts/target species selected on criteria that identify the ability to invade and become harmful; demonstrated impacts on environment, economy, human health, property, resources; strength and type of ecological interactions, e.g., ecological engineers; current distribution within biogeographic region and in other biogeographic regions; relationship with ballast water as a vector.	◗◯	▴□
	4. *Known impact and the assessment of the potential future impact on related ecosystem services.*		
	5. *Known impact and the assessment of potential future impact on human health, safety and the economy /* records of native species have the potential to affect human health, result in ecological, economic impacts/species of concern that may impair or damage the environment, human health, property or resources, target species should be selected for a specific port, State, or geographical region.	◗◯	▴□
	*Potential costs of damage (Art 5(1) (g))*		
	1. *The assessment, in monetary or other terms, of the potential costs of damage on biodiversity, ecosystem services.*	●	
	2. *The assessment of the potential costs of damage on human health, safety, and the economy.*	●	
	*Known uses for the species, social, economic benefits (Art 5(1) (h))*		
	1. *Description and list of known uses of species.*	●	
	2. *Social and economic benefits from the known uses for the species, environmental, social and economic relevance and an indication of associated beneficiaries.*	●	
Types of impact categories	*Human health*/Human health	◗◯	□
	*Economy*/Economy	◗◯	□
	*Environmental*/Environment	◗◯	□
	*Social –cultural*/Property or resources	◗◯	□

**Notes.**

a IMO (2007); European Union (2018).

b Precise definitions of the key principles are given in Table 1.

#### Key principles

The IMO Guidelines define eight key principles that should be taken into account in a RA. The EU Regulation mentions the RA principles, but not as explicit as the IMO guidelines. Consequently, our evaluation has been based on the key principles listed in the IMO Guidelines (Tables 1, 3).

#### RA components

The IMO Guidelines define three approaches of RA: (i) environmental matching, (ii) biogeographical and (iii) species-specific. All of these approaches are reflected in each of the eight articles outlining RA components in the EU Regulation. However, the IMO guidelines do not directly correspond with the two equivalent articles within the EU Regulation (*Art 5(1)(g)* and *Art 5(1)(h))* (Table 3), and only partially refer to the six other articles. Furthermore, the EU Regulation gives a brief description of those components that need to be addressed in RA methodologies. In our study we included all twenty-nine RA components of the EU Regulation in an overall evaluation procedure (Table 3).

#### Impact categories

The IMO Guidelines mention four impacts: “*on environment, economy, human health, property or resources*” (IMO, 2007). The EU Regulations include five impacts “*on biodiversity and related ecosystem services, including on native species, protected sites, endangered habitats, as well as on human health, safety, and the economy including an assessment of the potential future impact*” (European Union, 2018). The impacts referred to in both documents can be narrowed to four types: (a) human health, (b) economy, (c) environment (incl. biodiversity and ecosystem services), and (d) social-cultural values.

### Key principles of assessment process

The summary of the evaluation of compliance with the key principles is presented in Table 4 and detailed evaluation results are given in Table S3.

**Table 4:** Compliance of RA methods with key principles. “1” means that the method complies with key principle, according their criteria; 0 the method is not designed to cover key principle and their criteria.

Key principles	Bioinvasion risk and impact assessment methods (%)														
	AS-ISK	BINPAS	CIMPAL	CMIST	GABLIS	GB NNRA	GEIAA	GISS	GISS IUCN	HARMONIA+	GLOTSS	RABW	SBRA	TRAAIS	WISC
Effectiveness	1	1	1	1	1	1	1	1	1	1	1	1	1	1	1
Transparency	1	1	1	1	1	1	1	1	1	1	1	1	1	1	1
Consistency	1	0	0	1	0	0	0	1	0	1	0	0	0	0	0
Comprehensiveness	0	0	0	0	0	1	0	0	0	0	0	1	1	0	0
Risk management	1	1	0	1	1	0	0	1	1	1	1	1	1	1	1
Precautionary	1	1	1	1	0	1	1	1	1	1	1	1	1	1	1
Science based	1	1	1	1	1	1	1	1	1	1	1	1	1	1	1
Continuous improvement	1	1	1	1	0	1	0	1	0	1	0	1	1	0	1
*Coverage (%)*	***88***	***75***	***63***	***88***	***50***	***75***	***50***	***88***	***63***	***88***	***63***	***75***	***88***	***63***	***75***

#### Effectiveness

All methods complied with this principle and provided definitions of each parameter used and, included basic information as to how the assessment process could be undertaken.

#### Transparency

Was adequately addressed in three methods (BINPAS, CMIST, HARMONIA+). These tools are freely available as online information systems (Table S3). Other methods, while compliant with this principle, were less developed in this respect. Some methods (e.g., GB NNRA, GEIAA) provided either fully or in part through an available online service with an option to enter results to an online database. A further group of methods (e.g., AS-ISK, CIMPAL, GABLIS) based on case studies in the scientific literature, but these do not store results in an available database.

#### Consistency

According to the published data there were only four methods (AS-ISK, CMIST, GISS, HARMONIA+) we were able to examine for consistency, i.e., for repeatability of the test outcomes (Table S2). Such consistency was evaluated based on either expert judgment (e.g., D’hondt et al., 2015) or statistical scrutiny (Drolet et al., 2016). All remaining methods were considered to be non-compliant with the “consistency” principle as no relevant available published results found.

#### Comprehensiveness

Three methods complied with this principle (e.g., GB NNRA, SBRA, RABW) that considered all four bioinvasion impacts, i.e., human health (HH), economic (EC), environmental (EN) and social –cultural (SC). Three other methods (AS-ISK, GABLIS and HARMONIA+) considered EN, EC, SC, and a further three (TRAAIS, WISC and GISS) only two impacts EN, EC, while all other methods considered just environmental impacts.

#### Risk management

The majority of the methods (12 out of 15) fully addressed the “risk management” key principle by providing rankings of impact magnitude that could be used for making risk management decisions.

#### Precautionary

Fourteen out of fifteen methods fully addressed this principle and provided confidence levels for a final score and how to define uncertainty. Two methods (GB NNRA and GEIAA) incorporated levels of confidence for all risk assessment steps, but did not deal with levels of uncertainty. One method (GABLIS) did not provide any level of uncertainty or a confidence level.

#### Science based

All methods either complied fully, or in part, with justifying statements based on either experimental, field studies, or literature reviews.

#### Continuous improvement

Ten of the methods had been updated as in the case of AS-ISK (Copp et al., 2016), which evolved from the first version of FISK (Copp et al., 2009), while the original design was based on the Weed Risk Assessment methodology (Pheloung, Williams & Halloy, 1999). Two methods (BINPAS and CMIST) have been computerized following a theoretical background (Olenin, Minchin & Daunys, 2007 and IASWG, 2009) in order to provide an opportunity for online application (Narščius et al., 2012; Drolet et al., 2016).

### Risk assessment components

Based on the analysis we found that all methods incorporated at least some general information about non-indigenous species under consideration (Table 5; Table S1), i.e., taxonomic identity, scope of a RA, etc. (*Art 5 (1) (a*) (EU, 2018). The RA components concerning reproduction and spread (*Art 5 (1) (b*)), pathways (*Art 5 (1) (c*)), stages of invasion process (*Art 5 (1) (d*)), distribution (*Art 5 (1) (e*)) and impacts (*Art 5 (1) (f*)) were incorporated within most methods (Table 5). The least covered components were the estimated consequences of economic damage (*Art 5(1)(g)*) and any known uses and benefits (*Art 5(1)(h)*). This involved four and two methods, respectively.

**Table 5:** Incorporation of the RA components and their elements into the selected methods (%). The total number of elements in each RA component indicated in brackets.

RA components^a^	Relative proportion of RA elements (%) in the methods														
	AS-ISK	BINPAS	CIMPAL	CMIST	GABLIS	GB NNRA	GEIAA	GISS	GISS IUCN	HARMONIA+	GLOTSS	RABW	SBRA	TRAAIS	WISC
General information (5)	100	100	80	60	100	60	100	100	100	100	80	100	80	100	80
Reproduction and spread (3)	100	33	67	100	100	100	100	0	0	100	67	100	100	67	67
Pathways (7)	71	0	86	29	71	100	14	0	57	100	57	86	100	100	100
Stages of invasion process (3)	67	33	67	67	67	100	67	33	0	100	67	67	67	67	67
Distribution (2)	50	50	50	100	100	100	50	50	50	50	100	100	100	0	50
Impacts (5)	80	60	100	60	80	100	60	80	40	80	60	60	80	80	80
Potential costs of damage (2)	0	0	0	0	0	50	50	0	0	0	0	0	100	0	100
Known uses and benefits (2)	50	0	0	0	50	0	0	0	0	0	0	0	100	0	0
*Coverage (%)*	*72*	*38*	*69*	*52*	*76*	*83*	*55*	*38*	*41*	*79*	*59*	*72*	*90*	*72*	*76*

**Notes.**

a Additional information of RA components, elements and details of the analysis are in Table S4, for the methods see Table 2.

The incorporation of a RA component into a method was considered as being complete, should all of the elements be covered. For example, all three elements for reproduction and spread were incorporated within eight methods (Table S1). Only one method (SBRA) incorporated in full or in part all the components, while four methods (GABLIS, GB NNRA, HARMONIA+, WISC) incorporated more than 75% of the RA components.

### Types of impact

**Figure 2:**
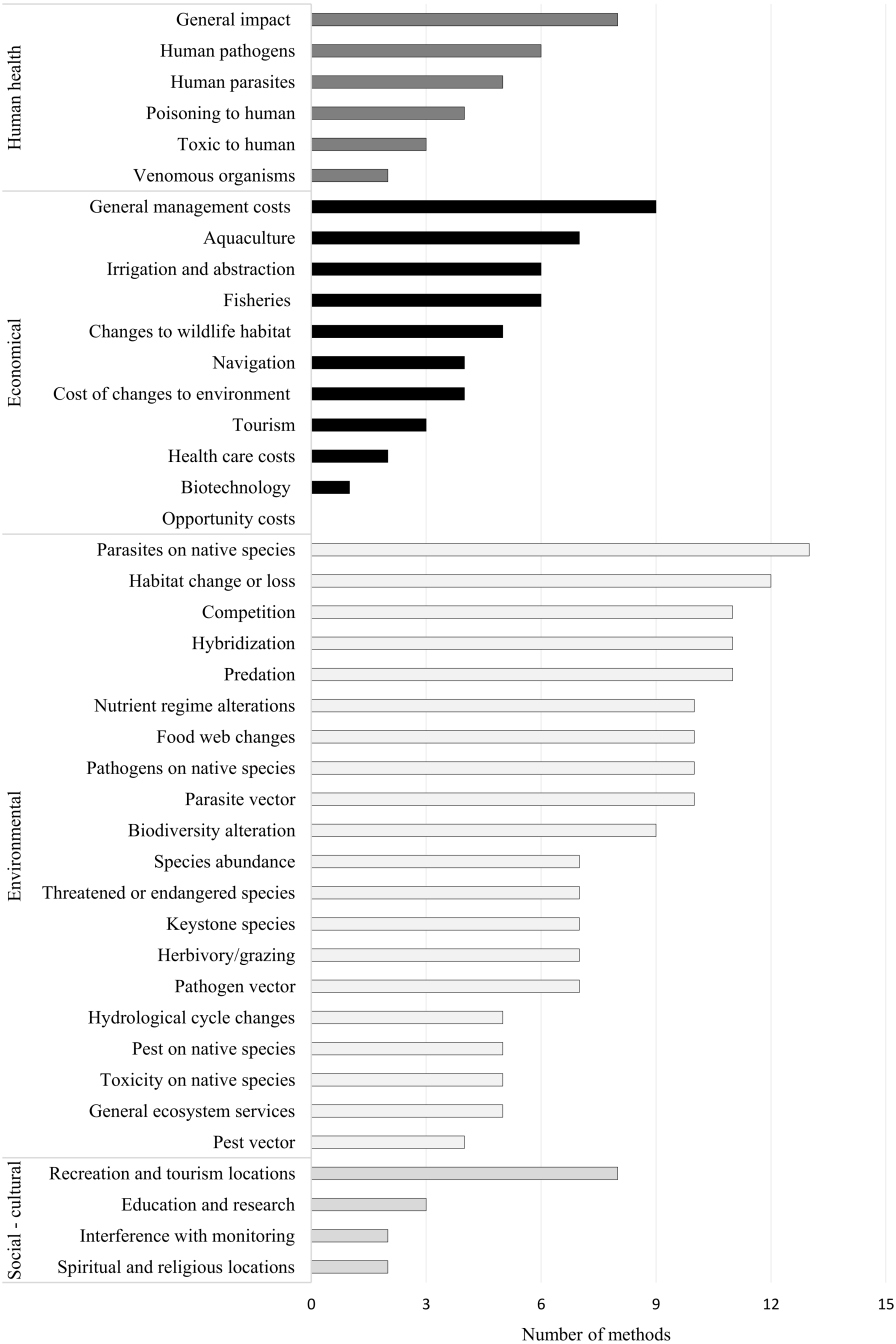
Comparison of categories with impact types in RA methods. The scale indicates the number of methods with corresponding categories of impact types.

The impacts on human health were considered in 57% of the methods, however, this was mostly as a “general impact on human health”, without further clarification. While three methods (GB NNRA, GISS, HARMONIA+) included more detailed information on human health, accordingly: parasites, pathogens, toxic compounds, poisoning and venomous organisms. These results are summarized in Table 6, Fig. 2 and additional information in Table S4.

**Table 6:** Summary of incorporation of types of impacts and their categories into the selected methods. Total number of categories in each types of impact indicated in brackets.

Types of impact	Relative proportion of types of impacts categories (%) in the methods														
	AS-ISK	BINPAS	CIMPAL	CMIST	GABLIS	GB NNRA	GEIAA	GISS	GISS IUCN	HARMONIA+	GLOTSS	RABW	SBRA	TRAAIS	WISC
Human health (6)	33	0	17	0	50	50	0	100	0	50	0	17	67	33	50
Economy (11)	46	0	9	0	55	36	0	64	0	36	36	9	64	27	46
Environment (20)	60	65	50	35	60	50	45	90	60	60	45	20	75	80	35
Social –cultural (4)	50	0	25	25	0	25	0	50	0	0	25	0	75	50	50
*Coverage (%)*	***51***	***32***	***32***	***20***	***51***	***44***	***22***	***80***	***29***	***46***	***34***	***15***	***71***	***56***	***41***

No method included all environmental impact categories. However, all had at least one environmental impact category: parasites and pathogens affecting native species, parasite vector, predation, competition, hybridization, habitat change, or population loss caused by an invasive species, etc. A single method (GISS) incorporated 90% of all of the environmental categories, followed by TRAAIS (80%) and SBRA (75%).

Sixty per-cent of the methods included an economic impact category with either general management costs (60%) or impacts to aquaculture (47%), fisheries (40%) or in relation to irrigation and abstraction (40%). The methods GABLIS, GISS, and SBRA covered more than a half of these economic categories: 55, 64 and 64%, respectively.

Social-cultural impacts were taken into account by 53% of methods, the most frequent category being consequences for recreation and tourism (53%). SBRA took into account seventy-five per-cent of the social-cultural impact categories, the highest coverage of any RA method.

### Overall evaluation of the methods

**Figure 3:**
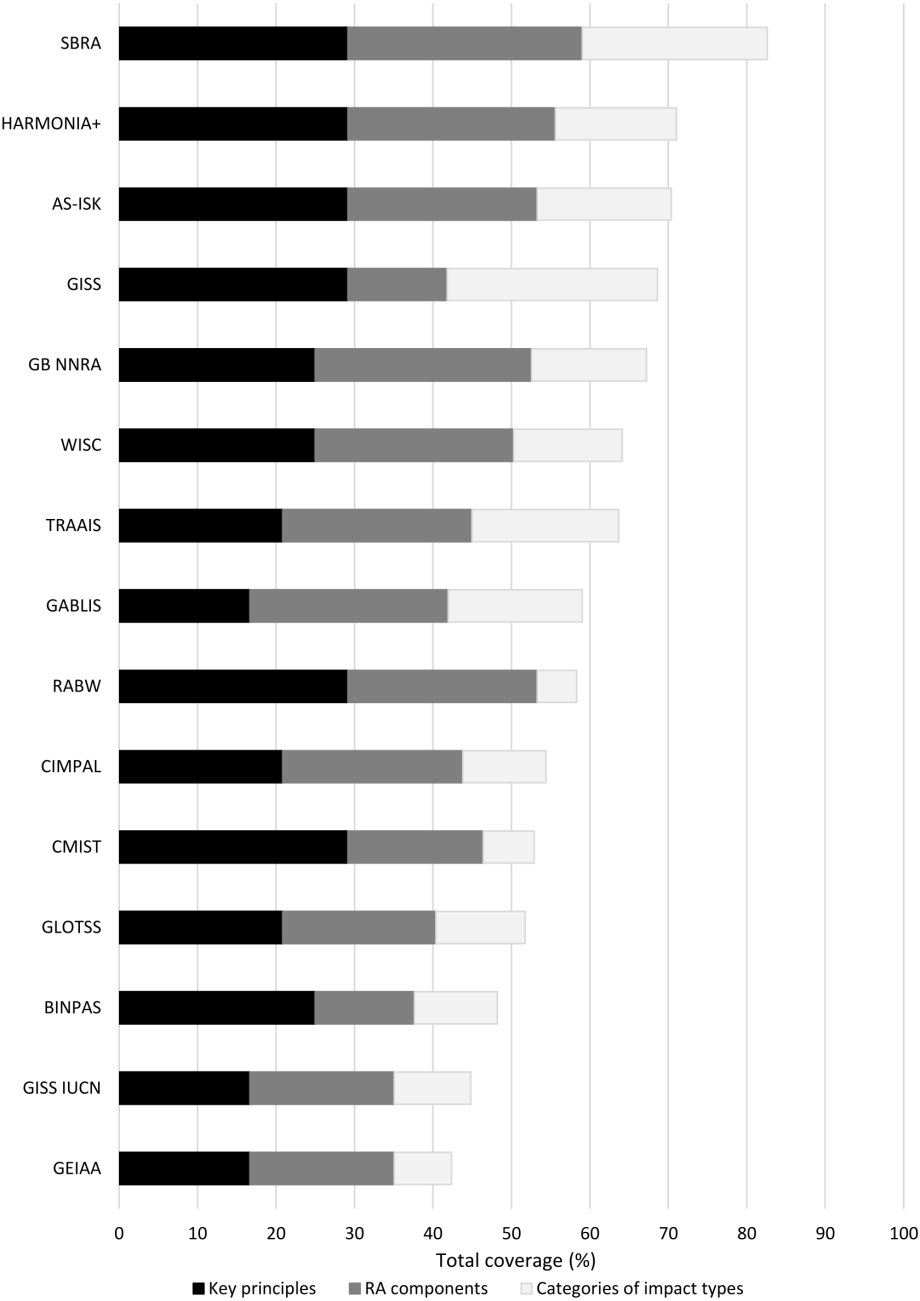
Overall compliance of the methods based on key principles, components and categories of impact types. Each comparison element: “key principles”, “RA components”, “types of impact categories” used in RA method expressed as a cumulative coverage (%).

Our general assessment of the methods by the key principles, RA components and categories of impacts are presented in Fig. 3, while the assessment of the methods according to criteria appears in Fig. S1. The method that met most of our criteria was SBRA. This complied with most of the key principles and RA components, and covered the broadest spectrum of the impact categories, followed by HARMONIA+ and AS-ISK. However, none of the methods complied with all our criteria.

A further method that generally complied well with key principles and RA components was RABW, a method developed for the BWMC. However, it has fewer impact categories, focusing only on those associated with aquatic environments. In contrast, GISS incorporates the highest number of impact categories, but has comparatively low compliance with RA components.

## Discussion

### Key principles and quality of methods

The two legislative documents reviewed in this study were developed for different purposes: while the EU regulation (European Union) has a wide spectrum of application and addresses the invasive alien species of all taxa and within all habitats, the IMO Guidelines (IMO) focuses on harmful aquatic organisms and pathogens transferred by a single vector of introduction. However, the cross-comparison of both documents highlighted the common features that stem from their overall orientation on biosecurity. Such comparative analysis is especially needed nowadays when EU countries are to implement both legally binding instruments, the BWMC (IMO, 2004), which entered into force in 2017 (IMO, 2017) and the EU the Regulation on the prevention and management of the introduction and spread of invasive alien species (Union European, 2014).

The comparison of the related risk assessment frameworks (IMO, 2007 and European Union, 2018) helps to achieve a more comprehensive, integrative view on the risk assessment process. As the result, the approach developed in this study is based on three criteria. Of these, the key principles and assessment components form the basic criteria in risk assessment, while the categories of the bioinvasion impacts were complimentary and added to complete the full evaluation procedure. This is because such impacts were not specified in either document.

The screening of similar regional and international regulations and frameworks, e.g., Convention on Biological Diversity (CBD, 2011), North America Free Trade Agreement—(General Accounting Office , GAO), Asia-Pacific Economic Cooperation (Williamson et al., 2002), ICES Code of Practice on the Introductions and Transfer of marine organisms (2005) did not reveal different criteria to what we have examined. Barry et al. (2008), Dahlstrom, Hewitt & Campbell (2011) and David & Gollasch (2015) who analyzed biosecurity risk assessment regulatory documents and bioinvasion risk and impact assessment methods also did not reveal criteria other than what we have used. It would seem that the key principles and RA components are universal for evaluation of bioinvasion risk and methods. The categories of the bioinvasion impacts may vary depending on the scope of the assessment and should be used as complimentary criteria.

In our opinion, the compliance with the key principles shows the quality of a method. Our analysis showed that only three methods made their assessment tools and documentation available *via* an online database. This must be considered the highest “Transparency” level and an example for other methods to follow. This is because decision-makers should have access to the full information to be able to compare the usage of bioinvasion risks and impacts assessment methods in similar situations worldwide.

Online information sources for NIS already exist for specific areas, e.g., for prioritizing most impacting NIS (e.g., target species lists), defining their pathways and vectors and with recommendations for their management (Olenin et al., 2014). Lehtiniemi et al. (2015) have stated that there is little value in monitoring of NIS unless the knowledge obtained is timely and can be directly used. The importance of dissemination of information, the transparency principle, was stressed many times at international and national levels (e.g., Awad et al., 2014; Costello et al., 2014; Sing & Tan, 2018). In addition, the availability of the information is important to achieve “a uniform high level of performance, using a common process and methodology” (IMO, 2007).

### Policy relevance of the methods

All bioinvasion risk and impact assessment methods reviewed here have been designed to support management decisions in a manner consistent with recommendations from multiple publications, e.g.: the method allows “a comparison and thus a prioritization of species” (Nentwig, Kühnel & Bacher, 2010), “enables an effective priorization of management efforts” (Sandvik et al., 2013), “identification of hotspots areas, and prioritization of sites, pathways and species for management actions” (Katsanevakis, Tempera & Teixeira, 2016), “inform management and policy decisions” (Drolet et al., 2016). Consequently, the methods should conform with the policy documents involved. In our study, the policy relevance may be defined as usefulness of a method for those who make decisions on biosecurity.

Our approach may help in choosing the most appropriate method, for example, to test the policy relevance of a method for the implementation of the BWMC. While only one method purposefully designed for the BWMC was analyzed in our study (RABW), several other methods may be used for the BWMC purposes with adjustment, should they follow the key principles and take into account RA components. For example, the GB NNRA method covers the components such as NIS spread, pathways, distribution and impacts, which are needed when considering a risk assessment of ballast water (Behrens, Leppäkoski & Olenin, 2005; Werschkun et al., 2014; Olenin et al., 2016). It is noteworthy that the method integrating most of the RA components (SBRA) was especially designed for one of the shipping vectors, i.e., for the species biofouling risk assessment (Hewitt et al., 2011). Ideally, all methods should comply with the key principles and RA components as far as possible, while the bioinvasion impact categories may vary and should be selected according to the purpose of the RA. For example, the risk to human health is an important issue (Conn, 2014); however, not all methods, even those purposefully designed for BWMC, take into account this impact category. In the earlier study by Barry et al. (2008) only two out of the eight reviewed “ballast water risk assessment systems” refer to the importance of human health categories without considering any details. Generally, our study has shown that more attention is paid to environmental impacts rather than to human health or economic impacts.

## Conclusion

Our study has shown that the IMO Guidelines and EU Regulation provide a common view of risk assessment process. The EU Regulation provides a broader coverage of the RA components and, in principle, incorporates all three IMO Guidelines requirements. This includes data needs for approaches towards environmental matching, biogeographical and species-specific matters. The common procedure developed for the evaluation of the bioinvasion risk and impact assessment methods includes a scoring scheme to assess compliance with the key principles, RA components and categories of bioinvasion impacts. It may be recommended for future methods, especially those designed for management of ballast water, to incorporate the EU Regulations RA components in addition to those recommended by the IMO Guidelines. Concerning the categories of the bioinvasion impacts, more attention should be paid to the impacts on human health and economy.

##  Supplemental Information

10.7717/peerj.6965/supp-1 Figure S1 The total coverage (%) of RA methods The graph scale indicates the percentage of total coverage of each method compliance with the key principles and RA components, and impact types categories. List of impact types is presented in Table 6, key principles and RA components Tables 4 and 5.

10.7717/peerj.6965/supp-2 Table S1 Screening results of compliance of bioinvasion risk and impact assessment methods together with the risk assessment components Data in the table shows the compliance of bioinvasion risk and impact assessment methods together with the risk assessment components and their elements. The elements derived from the EU regulation supplement on RA of IAS (EU, 2018) and IMO guidelines (IMO, 2007) are denoted as Risk Assessment (RA) components. “1” –indicates the presence, “0” –indicates the absence of common elements considered in bioinvasion risk and impact assessment methods. IMO RA approach types: ■—environmental matching risk assessment; ▴—species biogeographical risk assessment; □—species-specific risk assessment (IMO, 2007). The total number of RA component elements shows the number of components that are compliant with bioinvasion risk and impact assessment methods. The total coverage (%) –shows the relative proportion of all RA components (the percentage of components considered in the methods from all RA components). For the detailed information and a summary of the bioinvasion risk and impact assessment methods, see Table 2.

10.7717/peerj.6965/supp-3 Table S2 Descriptions of impact types categories Data in the table shows descriptions of categories and impact types (human health, economical, environmental, social –cultural), number of categories is given in brackets. A short description of each impact type categories and a list of literature is given.

10.7717/peerj.6965/supp-4 Table S3 Detailed description of compliance with the key principles of RA methods Data in the table shows the compliance of bioinvasion risk and impact assessment methods together with the key principles. The key principles (effectiveness, transparency, consistency, comprehensiveness, risk management, precautionary, science—based, continuous improvement) derived from the IMO guidelines (IMO, 2007). For the detailed information of key principles, see Table 1. For the detailed information and a summary of the bioinvasion risk and impact assessment methods, see Table 2.

10.7717/peerj.6965/supp-5 Table S4 Screening results for categories of impact types covered in methods Data in the table shows the compliance of bioinvasion risk and impact assessment methods together with the impact types (human health, economical, environmental, social –cultural) categories. “1” –indicates the presence, “0” –indicates the absence of impact types categories in bioinvasion risk and impact assessment methods. The total coverage (%) –shows the relative proportion of all impact types categories (the percentage of impact types categories considered in the methods from all impact types categories). The impact types categories derived from the literature, for the detailed information, see Table S3. For the detailed information and a summary of the bioinvasion risk and impact assessment methods, see Table 2.

## Additional Information and Declarations

### Data Availability

The following information was supplied regarding data availability: The raw data is available in Table 3 and the Supplemental Files.
