# Supplementary figures and images for: A comparison of impact and risk assessment methods based on the IMO Guidelines and EU invasive alien species risk assessment frameworks
Source: PeerJ. 2019 Jun 10;7:e6965. doi: 10.7717/peerj.6965 (PMC6563794; doi:10.7717/peerj.6965)

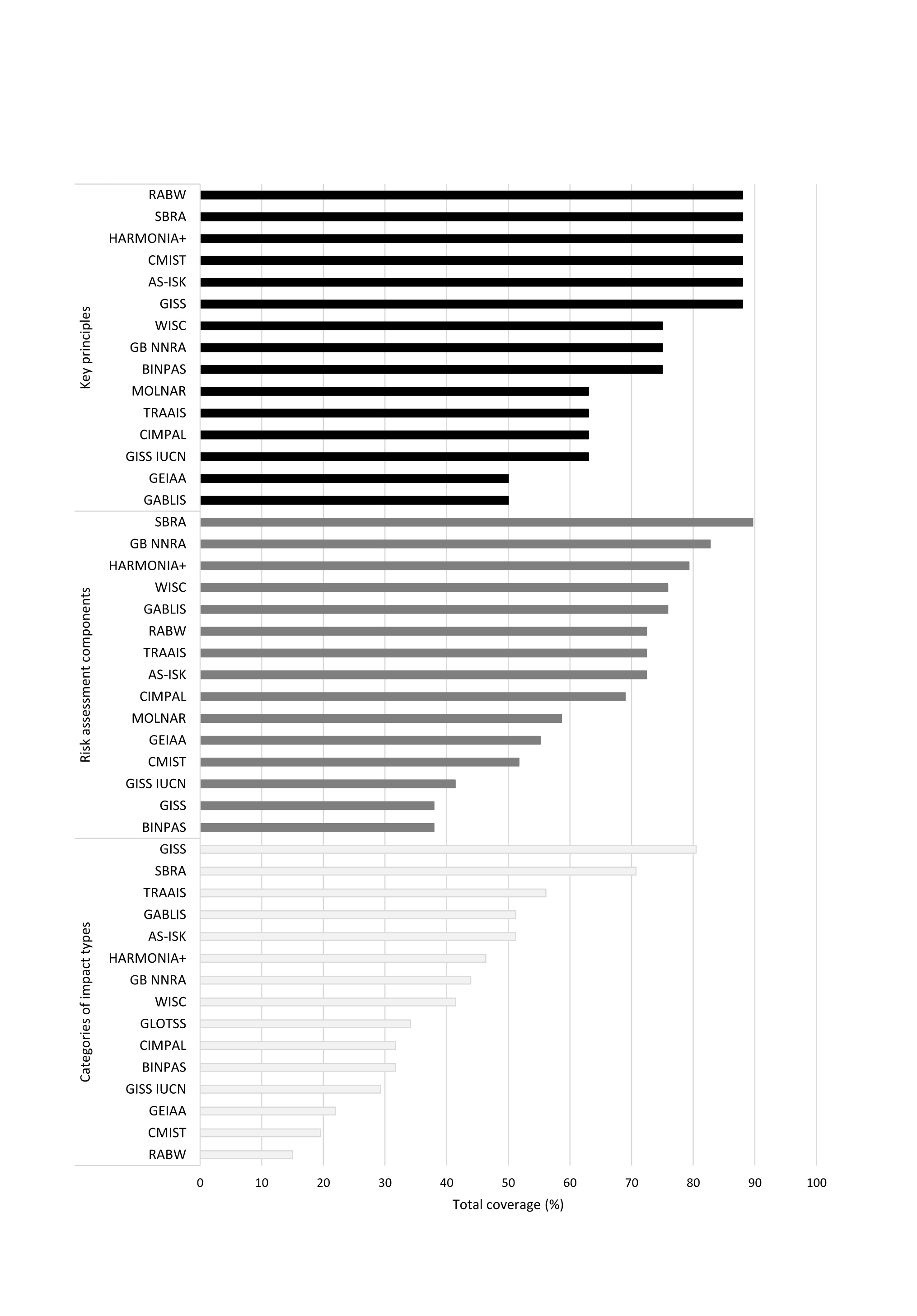

Supplement: Figure S1 — The graph scale indicates the percentage of total coverage of each method compliance with the key principles and RA components, and impact types categories. List of impact types is presented in Table 6, key principles and RA components Tables 4 and 5. [file peerj-07-6965-s001.png]
