# Supplementary material for: A comparison of impact and risk assessment methods based on the IMO Guidelines and EU invasive alien species risk assessment frameworks
Source: PeerJ. 2019 Jun 10;7:e6965. doi: 10.7717/peerj.6965 (PMC6563794; doi:10.7717/peerj.6965)
Supplement: Table S1 — Data in the table shows the compliance of bioinvasion risk and impact assessment methods together with the risk assessment components and their elements. The elements derived from the EU regulation supplement on RA of IAS (EU, 2018) and IMO guidelines (IMO, 2007) are denoted as Risk Assessment (RA) components. “1” –indicates the presence, “0” –indicates the absence of common elements considered in bioinvasion risk and impact assessment methods. IMO RA approach types: ■—environmental matching risk assessment; ▴—species biogeographical risk assessment; □—species-specific risk assessment (IMO, 2007). The total number of RA component elements shows the number of components that are compliant with bioinvasion risk and impact assessment methods. The total coverage (%) –shows the relative proportion of all RA components (the percentage of components considered in the methods from all RA components). For the detailed information and a summary of the bioinvasion risk and impact assessment methods, see Table 2. [file peerj-07-6965-s002.docx]

| RA components | Common elements described in EU regulation supplement on RA of IAS (EU, 2018) | IMO RA approach  type | Bioinvasion risk and impact assessment methods | | | | | | | | | | | | | | |
| --- | --- | --- | --- | --- | --- | --- | --- | --- | --- | --- | --- | --- | --- | --- | --- | --- | --- |
|  |  |  | **AS-ISK** | **BINPAS** | **CIMPAL** | **CMIST** | **GABLIS** | **GB NNRA** | **GEIAA** | **GISS** | **GISS IUCN** | **HARMONIA+** | **TRAAIS** | **SBRA** | **GLOTSS** | **WISC** | **RABW** |
| General information | **Art 5(1) (a) - a description of the species with its taxonomic identity, its history, and its natural and potential range** |  | **5/5** | **5/5** | **4/5** | **3/5** | **5/5** | **3/5** | **5/5** | **5/5** | **5/5** | **5/5** | **5/5** | **4/5** | **4/5** | **4/5** | **5/5** |
|  | 1. Description of species |  | 1 | 1 | 1 | 1 | 1 | 1 | 1 | 1 | 1 | 1 | 1 | 1 | 1 | 1 | 1 |
|  | 2. The scope of the risk assessment shall be clearly delineated |  | 1 | 1 | 1 | 1 | 1 | 1 | 1 | 1 | 1 | 1 | 1 | 1 | 0 | 1 | 1 |
|  | 3. The description of the taxonomic identity of the species |  | 1 | 1 | 1 | 1 | 1 | 1 | 1 | 1 | 1 | 1 | 1 | 1 | 1 | 1 | 1 |
|  | 4. The description of the history of the species | **■**▲**□** | 1 | 1 | 0 | 0 | 1 | 0 | 1 | 1 | 1 | 1 | 1 | 1 | 1 | 0 | 1 |
|  | 1. Information on countries invaded |  | + | - | - | - | + | - | + | + | + | + | + | + | + | - | + |
|  | 1. Indication of the timeline of the first observations |  | + | + | - | - | - | - | - | - | + | - | + | - | - | - | - |
|  | 1. Establishment and spread |  | + | - | - | - | - | - | - | - | - | + | + | - | - | - | - |
|  | 5. The description of the natural and potential range of the species | **■□** | 1 | 1 | 1 | 0 | 1 | 0 | 1 | 1 | 1 | 1 | 1 | 0 | 1 | 1 | 1 |
| Reproduction and spread | **Art 5(1) (b)- a description of its reproduction and spread patterns and dynamics including an assessment of whether the environmental conditions necessary for its reproduction and spread exist** |  | **3/3** | **1/3** | **2/3** | **3/3** | **3/3** | **3/3** | **3/3** | **0/3** | **0/3** | **3/3** | **2/3** | **3/3** | **2/3** | **2/3** | **3/3** |
|  | 1. The descriptions of reproduction and spread patterns | **■□** | 1 | 1 | 0 | 1 | 1 | 1 | 1 | 0 | 0 | 1 | 1 | 1 | 1 | 1 | 1 |
|  | 1. The species life history |  | + | + | - | + | + | + | + | - | - | + | + | + | - | - | + |
|  | 1. Behavioural traits |  | + | - | - | + | + | + | - | - | - | + | + | - | + | - | - |
|  | 1. Ability to establish and spread |  | + | - | - | + | + | + | + | - | - | + | + | + | + | + | + |
|  | 1. Reproduction or growth strategy |  | + | - | - | + | + | + | + | - | - | + | - | + | - | - | - |
|  | 1. Dispersal capacity |  | + | - | - | + | + | + | + | - | - | + | - | + | + | + | + |
|  | 1. Longevity |  | - | - | - | + | - | - | - | - | - | - | - | - | - | - | - |
|  | 1. Environmental and climatic requirements |  | + | - | - | - | + | + | - | - | - | + | - | + | - | - | + |
|  | 1. Specialist or generalist characteristics |  |  | - | - | + | - | - | - | - | - | - | - | - | - | - | - |
|  | *2.The description of the reproduction patterns and dynamics* | **■□** | 1 | 0 | 1 | 1 | 1 | 1 | 1 | 0 | 0 | 1 | 1 | 1 | 0 | 0 | 1 |
|  | 1. A list and description of the reproduction mechanisms |  | + | - | - | + | + | + | + | - | - | + | + | + | - | - | - |
|  | 1. Suitable environmental conditions for reproduction exist in the risk assessment area, e.g. number of gametes, eggs or propagules, reproductive cycles per year |  | - | - | + | - | - | + | + | - | - | + | - | - | - | - | - |
|  | 1. For each of those reproduction mechanisms in relation to the environmental conditions in the risk assessment area |  | + | - | - | - | + | + | - | - | - | + | - | + | - | - | + |
|  | *3. The description of the spread patterns and dynamics shall include all of the following elements* | **■□** | 1 | 0 | 1 | 1 | 1 | 1 | 1 | 0 | 0 | 1 | 0 | 1 | 1 | 1 | 1 |
|  | 1. Description of the spread mechanisms |  | + | - | - | + | + | - | + | - | - | + | - | + | + | - | - |
|  | 1. Suitable environmental conditions for the species' spread |  | + | - | + | + | + | + | - | - | - | + | - | + | - | + | + |
|  | 1. Rate of each of those spread mechanisms in relation to the environmental conditions |  | **+** | **-** | **-** | **-** | **+** | **+** | **-** | **-** | **-** | **+** | **-** | **+** | **-** | **+** | **+** |
| Pathways | **Art 5(1) (c) - a description of the potential pathways of introduction and spread of the species, both intentional and unintentional, including where relevant the commodities with which the species is generally associated** |  | **5/7** | **0/7** | **6/7** | **2/7** | **5/7** | **7/7** | **1/7** | **0/7** | **4/7** | **7/7** | **7/7** | **7/7** | **4/7** | **7/7** | **6/7** |
|  | *1. All relevant pathways for introduction as well as for spread* | ▲□ | 0 | 0 | 1 | 0 | 1 | 1 | 1 | 0 | 1 | 1 | 1 | 1 | 1 | 1 | 0 |
|  | *2. The description of intentional pathways of introduction* | ▲□ | 1 | 0 | 1 | 0 | 1 | 1 | 0 | 0 | 1 | 1 | 1 | 1 | 1 | 1 | 1 |
|  | 1. A list and description of pathways with an indication of their importance and associated risks (e.g. The likelihood of introduction into the risk assessment area; the likelihood of survival, reproduction or increase during transport and storage; the ability and likelihood of transfer, details about the specific origins and end points of the pathways) |  | + | - | + | - | + | + | - | - | + | + | + | + | + | + | + |
|  | 1. An indication of the propagule pressure (e.g. The estimated volume or number of specimens, or the frequency of passage through those pathways), including the likelihood of reinvasion after eradication. |  | - | - | - | - | - | - | - | - | - | - | + | + | - | - | - |
|  | *3. The description of unintentional pathways of introduction* | ▲□ | 1 | 0 | 1 | 0 | 1 | 1 | 0 | 0 | 1 | 1 | 1 | 1 | 1 | 1 | 1 |
|  | 1. A list and description of pathways with an indication of their importance and associated risks (e.g. The likelihood of introduction into the risk assessment area, the likelihood of survival, reproduction or increase during transport and storage; the likelihood of non-detection at the entry point; the ability and likelihood of transfer from those pathways to a suitable habitat or host), details about the specific origins and end points of the pathways |  | + | - | + | - | + | + | - | - | + | + | + | + | + | + | + |
|  | 1. An indication of the propagule pressure (e.g. The estimated volume or number of specimens, or the frequency of passage through those pathways) |  | - | - | - | - | - | - | - | - | - | - | + | + | - | - | - |
|  | *4. The description of commodities with which the introduction of the species is generally associated* | ▲□ | 0 | 0 | 1 | 0 | 1 | 1 | 0 | 0 | 1 | 1 | 1 | 1 | 1 | 1 | 1 |
|  | 1. A list and description of commodities with an indication of associated risks (e.g. The volume of trade flow; the likelihood of the commodity being contaminated or acting as a vector) |  | - | - | + | - | + | + | - | - | + | + | + | + | + | + | + |
|  | *5. The description of intentional pathways of spread* | ▲□ | 1 | 0 | 1 | 1 | 0 | 1 | 0 | 0 | 0 | 1 | 1 | 1 | 0 | 1 | 1 |
|  | 1. A list and description of pathways with an indication of their importance and associated risks (e.g. The likelihood of spread within the risk assessment area, based on those pathways; the likelihood of survival, reproduction or increase during transport and storage; the ability and likelihood of transfer from those pathways to a suitable habitat or host), including, where possible, details about the specific origins and end points of the pathways |  | + | - | + | - | - | + | - | - | - | + | + | + | - | + | + |
|  | 1. An indication of the propagule pressure (e.g. The estimated volume or number of specimens, or the frequency of passage through those pathways), including the likelihood of reinvasion after eradication |  | - | - | - | + | - | - | - | - | - | - | + | + | - | + | + |
|  | *6. The description of unintentional pathways of spread* | ▲□ | 1 | 0 | 1 | 0 | 0 | 1 | 0 | 0 | 0 | 1 | 1 | 1 | 0 | 1 | 1 |
|  | 1. A list and description of pathways with an indication of their importance and associated risks (e.g. The likelihood of spread within the risk assessment area, the likelihood of survival, reproduction or increase during transport and storage; the ease of detection; the ability and likelihood of transfer from those pathways to a suitable habitat or host), including details about the specific origins and end points of the pathways |  | + | - | + | - | - | + | - | - | - | + | + | + | - | + | + |
|  | 1. An indication of the propagule pressure (e.g. The estimated volume or number of specimens, or the frequency of passage through those pathways), including the likelihood of reinvasion after eradication |  | - | - | - | - | - | - | - | - | - | - | - | - | - | - | + |
|  | *7. The description of commodities with which the spread of the species is generally associated* | ▲□ | 1 | 0 | 0 | 1 | 1 | 1 | 0 | 0 | 0 | 1 | 1 | 1 | 0 | 1 | 1 |
|  | 1. The volume of trade |  | - | - | - | - | - | - | - | - | - | - | + | + | - | - | - |
|  | 1. The likelihood of a commodity being contaminated |  | + | - | - | + | + | + | - | - | - | + | + | + | - | - | + |
|  | 1. The likelihood of a commodity acting as vector |  | - | - | - | + | + | + | - | - | - | + | + | + | - | + | + |
| Stages of invasion process | **Art 5(1) (d) - a thorough assessment of the risk of introduction, establishment and spread in relevant biogeographical regions in current conditions and in foreseeable climate change conditions** |  | **2/3** | **1/3** | **2/3** | **2/3** | **2/3** | **3/3** | **2/3** | **1/3** | **0/3** | **3/3** | **2/3** | **2/3** | **2/3** | **2/3** | **2/3** |
|  | *1. The thorough assessment shall provide insights into the risks* | **■**▲**□** | 1 | 0 | 1 | 1 | 1 | 1 | 1 | 0 | 0 | 1 | 1 | 1 | 1 | 1 | 1 |
|  | 1. Insights into the risks of a species' introduction into relevant biogeographical regions in the risk assessment area |  | - | - | + | + | + | + | + | - | - | + | + | + | + | + | + |
|  | 1. Insights into the risks of a species establishment in relevant biogeographical regions in the risk assessment area |  | + | - | + | - | - | + | + | - | - | - | + | + | - | + | + |
|  | 1. Insights into the risks of a species spread within relevant biogeographical regions in the risk assessment area |  | + | - | + | - | - | + | + | - | - | - | + | + | - | + | + |
|  | *2. The thorough assessment of those risks does not have to include a full range of simulations* | **■**▲**□** | 0 | 0 | 0 | 0 | 0 | 1 | 0 | 0 | 0 | 1 | 0 | 0 | 0 | 0 | 0 |
|  | 1. Assessment of likely introduction, establishment and spread within a medium timeframe scenario (e.g. 30-50 years) with a clear explanation of the assumptions is provided. |  | - | - | - | - | - | + | - | - | - | + | - | - | - | - | - |
|  | *3. The risks referred to in point (1) may, for example, be described in terms of ‘likelihood’ or ‘rate’.* | **□** | 1 | 1 | 1 | 1 | 1 | 1 | 1 | 1 | 0 | 1 | 1 | 1 | 1 | 1 | 1 |
| Distribution | **Art 5(1) (e) description of the current distribution of the species, including whether the species is already present in the Union or in neighbouring countries, and a projection of its likely future distribution** |  | **1/2** | **1/2** | **1/2** | **2/2** | **2/2** | **2/2** | **1/2** | **1/2** | **1/2** | **1/2** | **1/2** | **2/2** | **2/2** | **1/2** | **2/2** |
|  | *1. The description of the current distribution in the risk assessment area or in neighbouring countries* | ▲**□** | 1 | 1 | 1 | 1 | 1 | 1 | 1 | 1 | 1 | 0 | 0 | 1 | 1 | 1 | 1 |
|  | *2. The projection of the likely future distribution in the risk assessment area or in neighbouring countries* | ▲**□** | 0 | 0 | 0 | 1 | 1 | 1 | 0 | 0 | 0 | 1 | 1 | 1 | 1 | 0 | 1 |
| Impacts | **Art 5(1) (f) description of the adverse impact on biodiversity and related ecosystem services, including on native species, protected sites, endangered habitats, as well as on human health, safety, and the economy including an assessment of the potential future impact having regard to available scientific knowledge** |  | **4/5** | **3/5** | **5/5** | **3/5** | **4/5** | **5/5** | **3/5** | **4/5** | **2/5** | **4/5** | **4/5** | **4/5** | **3/5** | **4/5** | **3/5** |
|  | *1. In the description, a distinction shall be made between the known impact and the potential future impact on biodiversity and related ecosystem services* | ▲**□** | 1 | 1 | 1 | 1 | 0 | 1 | 1 | 1 | 0 | 1 | 1 | 1 | 1 | 1 | 0 |
|  | 1. Biodiversity |  | + | + | + | + | + | + | + | + | - | + | + | + | + | + | - |
|  | 1. Ecosystem services |  | - | - | + | - | - | - | - | - | - | - | - | - | - | - | - |
|  | *2. The description of the known impact and the assessment of the potential future impact. The magnitude of the impact. The impact scoring or classification system.* | ▲ | 1 | 1 | 1 | 1 | 1 | 1 | 1 | 1 | 1 | 1 | 1 | 1 | 1 | 1 | 1 |
|  | 1. The description of the known impact and the assessment of the potential future impact. |  | + | + | - | - | + | + | + | + | + | + | + | + | + | + | + |
|  | 1. The magnitude of the impact. |  | + | + | + | + | + | + | + | + | + | + | + | + | - | + | - |
|  | 1. The impact scoring or classification system. |  | + | + | + | + | + | + | + | + | + | + | + | + | + | + | - |
|  | *3. The description of the known impact and the assessment of the potential future impact on biodiversity* | ▲**□** | 1 | 1 | 1 | 1 | 1 | 1 | 1 | 1 | 1 | 1 | 1 | 1 | 1 | 1 | 1 |
|  | 1. The different biogeographic regions or marine sub-regions where the species could establish |  | + | + | + | + | + | + | - | - | - | + | + | + | + | + | + |
|  | 1. Native species impacted, including red list species and species listed in the annexes of Council Directive 92/43/EEC (2) and species covered by Directive 2009/147/EC of the European Parliament and of the Council (3) |  | + | + | + | + | + | + | + | + | + | + | + | + | - | + | + |
|  | 1. Habitats impacted, including red list habitats and habitats listed in the annexes of Directive 92/43/EEC |  | - | + | + | + | + | + | + | + | + | + | + | + | + | - | + |
|  | 1. Protected sites impacted |  | - | - | + | + | - | + | + | + | + | + | + | + | - | + | + |
|  | 1. Impacted chemical, physical or structural characteristics and functioning of ecosystems |  | + | + | - | - | - | + | - | + | + | + | + | + | - | + | - |
|  | 1. Impacted ecological status of aquatic ecosystems or impacted environmental status of marine waters |  | - | + | - | - | - | + | - | - | - | - | - | - | - | - | - |
|  | *4. The description of the known impact and the assessment of the potential future impact on related ecosystem services* |  | 0 | 0 | 1 | 0 | 1 | 1 | 0 | 0 | 0 | 0 | 0 | 0 | 0 | 0 | 0 |
|  | 1. Provisioning services |  | - | - | + | - | - | + | - | - | - | - | - | - | - | - | - |
|  | 1. Regulating services, |  | - | - | + | - | + | + | - | - | - | - | - | - | - | - | - |
|  | 1. Cultural services |  | - | - | + | - | - | - | - | - | - | - | - | - | - | - | - |
|  | *5. The description of the known impact and the assessment of potential future impact on human health, safety and the economy* | ▲**□** | 1 | 0 | 1 | 0 | 1 | 1 | 0 | 1 | 0 | 1 | 1 | 1 | 0 | 1 | 1 |
|  | 1. Human health - illnesses, allergies or other affections to humans that may derive directly or indirectly from a species, |  | + | - | - | - | + | + | - | + | - | + | + | + | - | + | + |
|  | 1. Human safety - damages provoked directly or indirectly by a species with consequences for the safety of people, property or infrastructure |  | + | - | - | - | + | + | - | + | - | + | + | + | - | + | + |
|  | 1. Economy - direct or indirect disruption of, or other consequences for, an economic or social activity due to the presence of a specie |  | + | - | + | - | + | + | - | + | - | - | + | + | - | + | + |
| Potential costs of damage | **Art 5(1)(g) - an assessment of the potential costs of damage** |  | **0/2** | **0/2** | **0/2** | **0/2** | **0/2** | **1/2** | **1/2** | **0/2** | **0/2** | **0/2** | **0/2** | **2/2** | **0/2** | **2/2** | **0/2** |
|  | *1. The assessment, in monetary or other terms, of the potential costs of damage on biodiversity and ecosystem services* |  | 0 | 0 | 0 | 0 | 0 | 1 | 1 | 0 | 0 | 0 | 0 | 1 | 0 | 1 | 0 |
|  | *2. The assessment of the potential costs of damage on human health, safety, and the economy* |  | 0 | 0 | 0 | 0 | 0 | 0 | 0 | 0 | 0 | 0 | 0 | 1 | 0 | 1 | 0 |
| Known uses and benefits | **Art 5(1)(h) - a description of the known uses for the species and social and economic benefits deriving from those uses** |  | **1/2** | **0/2** | **0/2** | **0/2** | **1/2** | **0/2** | **0/2** | **0/2** | **0/2** | **0/2** | **0/2** | **2/2** | **0/2** | **0/2** | **0/2** |
|  | 1. The description of known uses for the species |  | 1 | 0 | 0 | 0 | 0 | 0 | 0 | 0 | 0 | 0 | 0 | 1 | 0 | 0 | 0 |
|  | 2. The description of social and economic benefits deriving from the known uses for the species |  | 0 | 0 | 0 | 0 | 1 | 0 | 0 | 0 | 0 | 0 | 0 | 1 | 0 | 0 | 0 |
| Total number of RA elements considered in the method/ Total number of RA elements | |  | **21/29** | **11/29** | **20/**  **29** | **15/29** | **22/29** | **24/29** | **16/29** | **11/29** | **12/29** | **23/29** | **21/29** | **26/29** | **17/29** | **22/29** | **21/29** |
| Total coverage (%) | |  | **72** | **38** | **69** | **52** | **76** | **83** | **55** | **38** | **41** | **79** | **72** | **90** | **59** | **76** | **72** |
