# Supplementary material for: A comparison of impact and risk assessment methods based on the IMO Guidelines and EU invasive alien species risk assessment frameworks
Source: PeerJ. 2019 Jun 10;7:e6965. doi: 10.7717/peerj.6965 (PMC6563794; doi:10.7717/peerj.6965)
Supplement: Table S2 — Data in the table shows descriptions of categories and impact types (human health, economical, environmental, social –cultural), number of categories is given in brackets. A short description of each impact type categories and a list of literature is given. [file peerj-07-6965-s003.docx]

| *Impact types*  Category | Description | Reference |
| --- | --- | --- |
| *Human health (6)* | | |
| Human pathogens | The organism is a pathogen and poses a threat to human health, consequences may be: mortality, illness, poisoning, toxicity, pain, irritation. Pathogens as *Vibrio cholerae, Escherichia coli,* and *Enterococci.* | AquaNIS. Editorial Board, 2019; Olenin et al., 2016; Vilà and Hulme 2017. |
| Human parasites | The organism is a parasite and poses a threat to human health; consequences could be: mortality, illness, poisoning, toxicity, pain, irritation. | Essl, 2011; Baker et al., 2008; Vilà and Hulme 2017. |
| Toxic | The organism has toxic compounds and poses a threat to human health, its flesh is toxic if consumed; organism produces chemical toxins which can be used to kill or incapacitate prey or as a defense against predators. | Katsanevakis et al., 2014; Essl et al., 2011; Vilà and Hulme 2017. |
| Poisoning | The organism is poisonous to humans; can decrease food security, worsen human health. | Copp et al., 2009; Copp et al., 2016; Nentwig et al., 2010; Vilà and Hulme 2017. |
| Venomous organisms | Venomous animals deliver toxins as venom through a bite, sting, or other specially evolved mechanisms. | Nentwig et al., 2010; Vilà and Hulme 2017. |
| General impact | General impact on human health, other risks (not mentioned above) to human health, to cause discomfort or pain to human, irritation, transmission of diseases, allergies, injuries, miscellaneous. | Copp et al., 2016; Essl, 2011; Mendoza et al., 2009; Nentwig et al., 2010; Vilà and Hulme, 2017. |
| *Environmental (20)* | | |
| Pest on native species | The organism is a pest and may have an impact on native species. | Copp et al., 2016; Nentwig et al., 2010; Mendoza et al., 2009; Vilà and Hulme, 2017. |
| Pathogen on native species | The organism is a pathogen and may have an impact on native species. | Olenin et al., 2007; Essl et al., 2011; Baker et al., 2008; Sandvik et al., 2013; Nentwig et al., 2010; Blackburn et al., 2014, D’hondt et al., 2015; Mendoza et al., 2009; Vilà and Hulme, 2017. |
| Parasite on native species | The organism is a parasite or poses parasitism on native species causing an impact on native species. | Olenin et al., 2007; Vilà and Hulme, 2017; Essl et al., 2011; Baker et al., 2008; Sandvik et al., 2013; Nentwig et al., 2010; Blackburn et al., 2014, D’hondt et al., 2015; Mendoza et al., 2009; Vilà and Hulme, 2017. |
| Pest vector | The organism may transfer their pest/pathogens/parasite (native or non-native) to the native species, which in turn disfavor native species and favors the invader itself. | Olenin et al., 2007; Essl et al., 2011; Baker et al., 2008; Sandvik et al., 2013; Nentwig et al., 2010; Blackburn et al., 2014, D’hondt et al., 2015; Mendoza et al., 2009; Vilà and Hulme, 2017. |
| Pathogen vector |  |  |
| Parasite vector |  |  |
| Habitat change or loss | The habitat alteration or loss, fragmentation and quality, type and size of affected habitat, reduced suitability.. | Olenin et al., 2007; Baker et al., 2008; Vilà and Hulme, 2017. |
| Biodiversity alteration | The alteration of biodiversity or richness has a negative or positive impact on native biota. | Manchester et al., 2000; Olenin et al., 2007; Katsanevakis et al., 2014; Essl et al., 2011; Baker et al., 2008; Nentwig et al., 2010; Mendoza et al., 2009; Vilà and Hulme, 2017. |
| Species abundance | General impact on native species abundance, decrease or increase of native species abundance. | Olenin et al., 2007; Katsanevakis et al., 2014; Sandvik et al., Nentwig et al., 2010; Vilà and Hulme, 2017. |
| Keystone species | General impact on keystone species abundance, decrease or increase of keystone species abundance. | Olenin et al., 2007; Essl et al., 2011; Baker et al., 2008; Sandvik et al., 2013; Nentwig et al., 2010; Blackburn et al., 2014, D’hondt et al., 2015; Mendoza et al., 2009; Vilà and Hulme, 2017. |
| Threatened or endangered species | General impact on threatened or endangered species abundance, decrease or increase of threatened species abundance. | Olenin et al., 2007; Essl et al., 2011; Baker et al., 2008; Sandvik et al., 2013; Nentwig et al., 2010; Blackburn et al., 2014, D’hondt et al., 2015; Mendoza et al., 2009; Vilà and Hulme, 2017. |
| Toxicity on native species | The organism has toxic compounds and poses a threat to native species. The organism produces chemical toxins which used to kill or incapacitate prey or as a defense against predators. | Olenin et al., 2007; Vilà and Hulme, 2017; Essl et al., 2011; Baker et al., 2008; Sandvik et al., 2013; Nentwig et al., 2010; Blackburn et al., 2014, D’hondt et al., 2015; Mendoza et al., 2009. |
| Predation | Predation refers to an interaction between two organisms, predator and prey, where there is a flow of energy from one to another. A process which causes declines. | Manchester et al., 2000; Olenin et al., 2007; Essl et al., 2011; Baker et al., 2008; Sandvik et al., 2013; Nentwig et al., 2010; Blackburn et al., 2014, D’hondt et al., 2015; Mendoza et al., 2009. |
| Herbivory/grazing | The organism feeds on plants (aquatic plants, benthic algae and phytoplankton) and/or sessile animals. | Manchester et al., 2000; Olenin et al., 2007; Essl et al., 2011; Baker et al., 2008; Sandvik et al., 2013; Nentwig et al., 2010; Blackburn et al., 2014, D’hondt et al., 2015; Mendoza et al., 2009. |
| Competition | The interaction between two or more organisms or species when both the organism or species are affected or harmed variously. | Manchester et al., 2000; Olenin et al., 2007; Essl et al., 2011; Baker et al., 2008; Sandvik et al., 2013; Nentwig et al., 2010; Blackburn et al., 2014, D’hondt et al., 2015; Mendoza et al., 2009. |
| Hybridization | The organism impacts genetic diversity and genetic structure of native species, genetic integrity of species, genetic constitution and phenotype. | Manchester et al., 2000; Olenin et al., 2007; Essl et al., 2011; Baker et al., 2008; Sandvik et al., 2013; Nentwig et al., 2010; Blackburn et al., 2014, D’hondt et al., 2015; Mendoza et al., 2009. |
| General ecosystem services | Changes to biological, chemical and physical properties of aquatic ecosystems. | Olenin et al., 2007; Essl et al., 2011; Baker et al., 2008; Sandvik et al., 2013; Nentwig et al., 2010; Blackburn et al., 2014, D’hondt et al., 2015; Mendoza et al., 2009. |
| Nutrient regime alteration | Increase or decrease of nutrient content, increased rates of nutrient mineralization. | Olenin et al., 2007; Essl et al., 2011; Baker et al., 2008; Sandvik et al., 2013; Nentwig et al., 2010; Blackburn et al., 2014, D’hondt et al., 2015; Mendoza et al., 2009. |
| Hydrological cycle changes | Changes to hydrographic regimes and topography. | Olenin et al., 2007; D’hondt et al., 2015; Mendoza et al., 2009. |
| Food web changes | Changes of the food web as a result of the addition or reduction of functional groups within trophic levels. | Olenin et al., 2007; Essl et al., 2011; Baker et al., 2008; Sandvik et al., 2013; Nentwig et al., 2010; Blackburn et al., 2014, D’hondt et al., 2015; Mendoza et al., 2009. |
| *Economical (11)* | | |
| Fisheries | Ecosystem that provides current or potential economic values, consumer reaction, alteration to fishery. | Manchester et al., 2000; Emerton and Howard 2008; A. Dahlstrom et al., 2011. |
| Aquaculture | Ecosystem that provides current or potential economic values. Gain or loss on farming aquatic organism. | Manchester et al., 2000; Emerton and Howard 2008. |
| Biotechnology | Changes in abundance of medical marine plants, algae and fisheries resources. | Emerton and Howard 2008. |
| Cost of changes to environment | Changes of environment causing economic consequences, loss of nursery areas, commercial species, commercially relevant infrastructure. | Manchester et al., 2000; Emerton and Howard 2008. |
| Navigation | Loss of access due to choking of waterways, changes to port infrastructure, canals, offshore wind and tidal generation, desalination plants. | Emerton and Howard 2008. |
| Changes to wildlife habitat | Impact on nature reserves, costs to remove or prevent non-indigenous species. | Manchester et al., 2000; Emerton and Howard 2008. |
| Irrigation and abstraction | Irrigation and abstraction of water canals, power plants, and municipal supplies. | Emerton and Howard 2008. |
| General management costs | Costs associated to additional research and administration, monitoring, quarantine. | Emerton and Howard 2008. |
| Tourism | Impact on tourism, touristic sites, decrease or increase of value touristic sites, hiker and ecotourism visitations. | Emerton and Howard 2008. |
| Health care costs | Costs associated to increase of health hazards risks which lead to economic loss. | Nentwig et al., 2010; Emerton and Howard 2008. |
| Opportunity costs | Opportunity costs approach may serve to justify accepting the largest, and environmentally damaging development projects. These costs can be explored via scenarios of future. | Crowards,1998; Turner et al., 2008. |
| *Social – cultural (4)* | | |
| Recreation and tourism locations | Symbolic‐aesthetic values for human usage for pleasure of a species or habitats. | Katsanevakis et al., 2014; Emerton and Howard 2008. |
| Spiritual proposes and religious locations | Species, locations, habitats used for spiritual proposes; iconic or spiritual value, including locations that create a sense of local, regional, or national identity. | Katsanevakis et al., 2014; Emerton and Howard 2008. |
| Education and research | Species, locations, habitats used for education and research. | Katsanevakis et al., 2014; Ojaveer et al., 2015; Emerton and Howard 2008. |
| Interference with monitoring | Interference with long-term or short-term monitoring. | Olenin et al., 2016; Emerton and Howard 2008. |
