# Supplementary material for: A comparison of impact and risk assessment methods based on the IMO Guidelines and EU invasive alien species risk assessment frameworks
Source: PeerJ. 2019 Jun 10;7:e6965. doi: 10.7717/peerj.6965 (PMC6563794; doi:10.7717/peerj.6965)
Supplement: Table S3 — Data in the table shows the compliance of bioinvasion risk and impact assessment methods together with the key principles. The key principles (effectiveness, transparency, consistency, comprehensiveness, risk management, precautionary, science—based, continuous improvement) derived from the IMO guidelines (IMO, 2007). For the detailed information of key principles, see Table 1. For the detailed information and a summary of the bioinvasion risk and impact assessment methods, see Table 2. [file peerj-07-6965-s004.docx]

| Bioinvasion risk and impact assessment methods | Key principles | | | | | | | |
| --- | --- | --- | --- | --- | --- | --- | --- | --- |
|  | Effectiveness | Transparency | Consistency | Compre-  hensiveness | Risk management | Precautionary | Science - based | Continuous improvement |
| AS-ISK | Use of an offline application, definitions of parameters are provided, the calculation scheme is described and clear, the result is obtained automatically. | The evidence supporting assessment is documented and available on request (n.o.). | The consistency was tested, results were published in peer-reviewed literature. (Copp et al., 2016) | Three categories (EN, EC, HH). | Categories of risk, from unacceptable to acceptable, management decisions included. | Level of confidence for each risk assessment step, and final score, clear instructions to define uncertainty is given. | The method based on quantitative and qualitative data, assesses biological traits, environmental tolerance and impacts. | The method has been updated since original version  (Pheloung et al., 1999; Copp et al., 2005; Tricarico et al., 2010). |
| BINPAS | Definitions of all parameters provided, the calculation scheme is clear, the result is obtained automatically using an *via* online system^1^ platform. | The evidence supporting assessment is documented, and available *via* online system^1^. | The assessment of the consistency was not available publicly. | One category (EN). | Five levels of risk (from minimum till high), except human health. | Level of confidence for each risk assessment step. | The method takes into account impacts, assess biological traits, part of assessment is based on quantitative and qualitative data. | The method has been updated since original version (Olenin et al., 2007) was computerized and renamed BINPAS/ Biopollution assessment system. |
| CIMPAL | Definitions of all parameters provided, the calculation scheme is clear, the result is obtained automatically. | The evidence supporting the assessment is not provided, available on request (n. o.). | The assessment of the consistency was not available publicly. | One category (EN). | Index defines the level of risk in general, sites specific value by environmental impact. | The ranking of sites based on the uncertainty-averse, the precautionary approach. | The method takes into account assessment of environmental tolerance limits and physical habitat characteristics. | The method has been updated since original version (Mazaris and Katsanevakis, 2018). |
| CMIST | Use of a method is supported by the questionnaire, definitions of parameters provided, the calculation scheme is clear, the result is obtained automatically. | The evidence supporting the assessment is documented and available *via* online system^2^. | The consistency of a method was tested (Drolet et al., 2016). | One category (EN). | The method defines final risk in general (except human health). | Incorporates level of confidence for all risk assessment steps. Clear instructions to define uncertainty is given, no confidence level for the final score. | The method assesses biological traits, environmental tolerance limits, assessment is based on quantitative field and experimental data. | The method has been updated since original version, is a modification of the Alberta Risk Assessment Tool (IASWG 2009; Drolet et al., 2014; Drolet et al., 2015;). |
| GABLIS | The method is represented by a questionnaire, the calculation scheme is clear. | The evidence supporting the assessment is documented and available on request (n. o.). | The assessment of the consistency was not available publicly. | Three categories (EN, EC, HH). | The method defines species into ranks, management decisions included. | No level of confidence is included. | Data from scientific reports, peer-reviewed publications, expert judgement used. Assess biological traits (indirectly), environmental tolerance limits. | Only original version exists, has no update published version (Essl et al., 2012) |
| GB NNRA | The method is represented by questionnaire available online. No calculation scheme is included, the assessment is qualitative. | The evidence supporting the assessment is documented and available *via* online system^3^. | The assessment of the consistency was not available publicly. | Four categories (EN, EC, HH, SC) | The method defines the categories of risk in general. | Level of confidence for each assessment steps and for the final risk score. | The method assesses biological traits, environmental tolerance limits, probability of entry, establishment, spread and impacts. | The method has been updated since original version.  (Baker et al., 2008; Mumford et al., 2010). |
| GEIAA | Definitions of parameters are provided, the calculation scheme is clear, the result is obtained automatically. | The evidence supporting the assessment is available *via* online system^4^. | The assessment of the consistency was not available publicly. | One category (EN) (detailed SD, CO, PRP, HP, TI, TG, PR, PH) | The method defines the categories of impact in general, five categories (except human health). | Incorporates level of confidence for each assessment step, but not for the final score. No detailed information for uncertainty. | The method assesses biological traits (generation time, expected population lifetime, expansion velocity). | Only original version exists, has no update published version (Sandvik et al., 2013) |
| GISS | The method is represented by a questionnaire. The calculation scheme is clear. | The evidence supporting the assessment is documented and available on request. | The consistency of a method was tested (Evans et al.,  2014). | Two categories (EN, EC, HH, HS, HI). | The method defines the level of risk, including risk to human health. | Incorporates level of confidence for all each assessment step and for the final score. | The method uses published information, does not includes the biological traits or environmental tolerance limits. | The method has been updated since original version (Nentwig et al., 2016, Blackburn et al., 2014) |
| GISS IUCN | The method is represented by a questionnaire. Definitions of parameters are provided, the calculation scheme is clear, the result is obtained automatically. | The evidence supporting the assessment are documented and available on request. | The assessment of the consistency was not available publicly. | One category (EN). | The method defines the categories of impact in general (except human health). | Incorporates level of confidence for all steps and for the final score. Clear instructions to define uncertainty is given. | The method assesses impacts, based on literature overview, does not includes biological traits or environmental tolerance limits. | Only original version exists, has no update published version. |
| HARMONIA+ | The method is available online, definitions provided, the calculation scheme is clear, the result is obtained automatically. | The evidence supporting the assessment is documented and available *via* online system^5^. | The consistency of a method was tested (D’hondt et al., 2015). | Three categories (EN, EC, HH, HI). | The method clearly defines the categories of risk, from unacceptable to acceptable, including decision making. | Incorporates level of confidence for all risk assessment steps, but not for the final score. | The method assesses biological traits, environmental tolerance limits, assessment is based on quantitative field and/or experimental data. | The method has been updated since original version (D’hondt et al., 2016). |
| TRAAIS | The method is represented by a questionnaire, the calculation scheme is clear. | The evidence supporting the assessment are documented and available on request. | The assessment of the consistency was not available publicly. | Two categories (EN, EC). | The method defines the categories of risk in general (possible threating categories, except human health), no level of risks described. | Incorporates level of confidence for all risk assessment steps, but not for the final score. | The method assesses biological traits, environmental tolerance limits, assessment is based on quantitative field data. | Only original version exists, has no update published version. |
| GLOTSS | The method is represented by a questionnaire, the calculation scheme is clear, the assessment is qualitative. | The evidence is documented, available *via* online system^6^. | The assessment of the consistency was not available publicly. | One category (EN) | The method defines the categories of risk in general, no level of risks described, management question included. | Incorporates level of confidence documentation source, but not for the final score. | Indirectly assess biological traits (invasive potential, spread), assessment is based on quantitative field and/or experimental data. | Only original version exists, has no update published version. |
| WISC | The method is represented by a questionnaire, the calculation scheme is clear, the assessment is qualitative. | The evidence supporting the assessment are documented and available on request. | The assessment of the consistency was not available publicly. | Two categories (EN, EC). | The method defines the categories of risk in general, level of risks described, management question included. | Incorporates level of confidence for each assessment step and for the final score. | Indirectly assess biological traits (potential for entry, spread), assessment is based on quantitative field and/or experimental data. | The method has been updated since original version, adapted from Alaska’s ranking system. |
| SBRA | The method is represented by a questionnaire, the calculation scheme is clear, the assessment is qualitative. | The evidence supporting the assessment are documented and available on request. | The assessment of the consistency was not available publicly. | Four categories (EN, EC, HH, SC). | The method defines the categories of risk in general, level of risks described, management question included. | Incorporates uncertainty for risk part of assessment. | Assess biological traits, environmental tolerance limits, assessment is based on quantitative field and/or experimental data. | The method has been updated since original version. |
| RABW | The method is represented by a questionnaire, the assessment is qualitative. | The evidence  supporting the assessment are documented and available on request. | The assessment of the consistency was not available publicly. | Four categories (EN, EC, HH, SC). | The method clearly defines the categories of risk, from unacceptable to acceptable, including decision making. | Incorporates level of confidence for all risk assessment steps. | Assess biological traits, environmental tolerance limits, assessment is based on quantitative field and/or experimental data. | The method has been updated since original version. |

Explanation: Environmental - EN, human health - HH, economic – EC, social and cultural – SC, HS – human social life, HI – human infrastructure; n. o. - not freely available online;

BINPAS via online system^1^ - <http://www.corpi.ku.lt/databases/index.php/binpas>; CMIST via online system^2^ - <http://www.bio.gc.ca/science/monitoring-monitorage/cmist/index-en.php>; GB NNRA *via* online system^3^ - <http://www.nonnativespecies.org/alerts/index.cfm>; GEIAA *via* online system^4^ - <https://www.artsdatabanken.no>; HARMONIA+ *via* online system^5^ - <https://ias.biodiversity.be/species/risk>; <http://ias.biodiversity.be>; GLOTSS - *via* online system^6^ - <http://conserveonline.org/workspaces/global.invasive.assessment>;
