# Supplementary material for: A comparison of impact and risk assessment methods based on the IMO Guidelines and EU invasive alien species risk assessment frameworks
Source: PeerJ. 2019 Jun 10;7:e6965. doi: 10.7717/peerj.6965 (PMC6563794; doi:10.7717/peerj.6965)
Supplement: Table S4 — Data in the table shows the compliance of bioinvasion risk and impact assessment methods together with the impact types (human health, economical, environmental, social –cultural) categories. “1” –indicates the presence, “0” –indicates the absence of impact types categories in bioinvasion risk and impact assessment methods. The total coverage (%) –shows the relative proportion of all impact types categories (the percentage of impact types categories considered in the methods from all impact types categories). The impact types categories derived from the literature, for the detailed information, see Table S3. For the detailed information and a summary of the bioinvasion risk and impact assessment methods, see Table 2. [file peerj-07-6965-s005.docx]

| Impact types  Categories | Bioinvasion risk and impact assessment methods | | | | | | | | | | | | | | |
| --- | --- | --- | --- | --- | --- | --- | --- | --- | --- | --- | --- | --- | --- | --- | --- |
|  | AS-ISK | BINPAS | CIMPAL | CMIST | GABLIS | GB NNRA | GEIAA | GISS | GISS IUCN | HARMONIA+ | TRAAIS | GLOTSS | WISC | SBRA | RABW |
| Human health | **2/6** | **0/6** | **1/6** | **0/6** | **3/6** | **3/6** | **0/6** | **6/6** | **0/6** | **3/6** | **2/6** | **0/6** | **3/6** | **4/6** | **1/6** |
| Human pathogen | 0 | 0 | 0 | 0 | 1 | 1 | 0 | 1 | 0 | 1 | 1 | 0 | 0 | 1 | 0 |
| Human parasites | 0 | 0 | 0 | 0 | 1 | 1 | 0 | 1 | 0 | 1 | 1 | 0 | 0 | 0 | 0 |
| General impact | 1 | 0 | 0 | 0 | 1 | 1 | 0 | 1 | 0 | 1 | 0 | 0 | 1 | 1 | 1 |
| Toxic to human | 0 | 0 | 1 | 0 | 0 | 0 | 0 | 1 | 0 | 0 | 0 | 0 | 0 | 1 | 0 |
| Poisoning to human | 1 | 0 | 0 | 0 | 0 | 0 | 0 | 1 | 0 | 0 | 0 | 0 | 1 | 1 | 0 |
| Venomous organisms | 0 | 0 | 0 | 0 | 0 | 0 | 0 | 1 | 0 | 0 | 0 | 0 | 1 | 0 | 0 |
| Economical | **5/11** | **0/11** | **1/11** | **0/11** | **6/11** | **4/11** | **0/11** | **7/11** | **0/11** | **4/11** | **3/11** | **4/11** | **5/11** | **7/11** | **1/11** |
| General management costs | 1 | 0 | 1 | 0 | 0 | 1 | 0 | 1 | 0 | 0 | 1 | 1 | 1 | 1 | 1 |
| Fisheries | 1 | 0 | 0 | 0 | 1 | 0 | 0 | 1 | 0 | 0 | 1 | 0 | 1 | 1 | 0 |
| Aquaculture | 1 | 0 | 0 | 0 | 1 | 0 | 0 | 1 | 0 | 0 | 1 | 1 | 1 | 1 | 0 |
| Changes to wildlife habitat | 1 | 0 | 0 | 0 | 1 | 1 | 0 | 1 | 0 | 0 | 0 | 0 | 1 | 0 | 0 |
| Cost of changes to environment | 1 | 0 | 0 | 0 | 0 | 1 | 0 | 0 | 0 | 1 | 0 | 0 | 0 | 1 | 0 |
| Irrigation and abstraction | 0 | 0 | 0 | 0 | 1 | 0 | 0 | 1 | 0 | 1 | 0 | 1 | 1 | 1 | 0 |
| Navigation | 0 | 0 | 0 | 0 | 1 | 0 | 0 | 1 | 0 | 0 | 0 | 1 | 0 | 1 | 0 |
| Tourism | 0 | 0 | 0 | 0 | 1 | 0 | 0 | 0 | 0 | 1 | 0 | 0 | 0 | 1 | 0 |
| Health care costs | 0 | 0 | 0 | 0 | 0 | 1 | 0 | 1 | 0 | 0 | 0 | 0 | 0 | 0 | 0 |
| Biotechnology | 0 | 0 | 0 | 0 | 0 | 0 | 0 | 0 | 0 | 1 | 0 | 0 | 0 | 0 | 0 |
| Opportunity costs | 0 | 0 | 0 | 0 | 0 | 0 | 0 | 0 | 0 | 0 | 0 | 0 | 0 | 0 | 0 |
| Environment | **12/20** | **13/20** | **10/20** | **7/20** | **12/20** | **10/20** | **9/20** | **18/20** | **12/20** | **12/20** | **16/20** | **9/20** | **7/20** | **15/20** | **4/20** |
| Parasite on native species | 1 | 1 | 1 | 1 | 1 | 1 | 1 | 1 | 1 | 1 | 1 | 0 | 0 | 1 | 1 |
| Predation | 1 | 1 | 1 | 1 | 1 | 1 | 0 | 1 | 1 | 1 | 1 | 0 | 0 | 1 | 0 |
| Hybridization | 1 | 1 | 0 | 1 | 1 | 1 | 1 | 1 | 1 | 1 | 1 | 0 | 1 | 0 | 0 |
| Parasite vector | 1 | 1 | 0 | 1 | 1 | 1 | 1 | 1 | 0 | 1 | 1 | 0 | 0 | 1 | 0 |
| Habitat change or loss | 1 | 1 | 1 | 1 | 0 | 1 | 1 | 1 | 1 | 0 | 1 | 1 | 1 | 1 | 0 |
| Competition | 1 | 1 | 1 | 1 | 1 | 0 | 0 | 1 | 1 | 1 | 1 | 1 | 0 | 1 | 0 |
| Pathogen on native species | 0 | 1 | 0 | 0 | 1 | 1 | 1 | 1 | 1 | 1 | 1 | 0 | 0 | 1 | 1 |
| Food web changes | 1 | 1 | 1 | 0 | 1 | 0 | 0 | 1 | 1 | 1 | 1 | 1 | 0 | 1 | 0 |
| Nutrient regime alterations | 1 | 1 | 0 | 0 | 1 | 1 | 0 | 1 | 1 | 1 | 1 | 1 | 0 | 1 | 0 |
| Biodiversity alteration | 0 | 1 | 1 | 0 | 1 | 1 | 0 | 1 | 0 | 0 | 1 | 1 | 1 | 1 | 0 |
| Pathogen vector | 0 | 0 | 0 | 0 | 1 | 1 | 1 | 1 | 0 | 1 | 1 | 0 | 0 | 1 | 0 |
| Herbivory/grazing | 0 | 0 | 1 | 0 | 1 | 0 | 0 | 1 | 1 | 1 | 1 | 0 | 0 | 1 | 0 |
| General ecosystem services | 1 | 0 | 0 | 1 | 0 | 1 | 0 | 1 | 1 | 0 | 0 | 0 | 0 | 0 | 0 |
| Keystone species | 0 | 1 | 1 | 0 | 0 | 0 | 1 | 0 | 0 | 1 | 1 | 1 | 1 | 0 | 0 |
| Threatened or endangered species | 1 | 0 | 0 | 0 | 0 | 0 | 1 | 1 | 0 | 1 | 1 | 1 | 1 | 0 | 0 |
| Toxicity on native species | 0 | 1 | 1 | 0 | 0 | 0 | 0 | 1 | 1 | 0 | 0 | 0 | 0 | 1 | 0 |
| Species abundance | 0 | 1 | 1 | 0 | 0 | 0 | 1 | 1 | 0 | 0 | 0 | 1 | 1 | 1 | 0 |
| Pest vector | 1 | 0 | 0 | 0 | 0 | 0 | 0 | 1 | 0 | 0 | 1 | 0 | 0 | 1 | 0 |
| Pest on native species | 1 | 0 | 0 | 0 | 0 | 0 | 0 | 1 | 0 | 0 | 1 | 0 | 0 | 1 | 1 |
| Hydrological cycle changes | 0 | 0 | 0 | 0 | 1 | 0 | 0 | 0 | 1 | 0 | 0 | 1 | 1 | 0 | 1 |
| Social and cultural | **2/4** | **0/4** | **1/4** | **1/4** | **0/4** | **1/4** | **0/4** | **2/4** | **0/4** | **0/4** | **2/4** | **1/4** | **2/4** | **3/4** | **0/4** |
| Recreation and tourism locations | 1 | 0 | 1 | 1 | 0 | 1 | 0 | 1 | 0 | 0 | 1 | 0 | 1 | 1 | 0 |
| Education and research | 0 | 0 | 0 | 0 | 0 | 0 | 0 | 1 | 0 | 0 | 1 | 0 | 1 | 0 | 0 |
| Spiritual and religious locations | 1 | 0 | 0 | 0 | 0 | 0 | 0 | 0 | 0 | 0 | 0 | 0 | 0 | 1 | 0 |
| Interference with monitoring | 0 | 0 | 0 | 0 | 0 | 0 | 0 | 0 | 0 | 0 | 0 | 1 | 0 | 1 | 0 |
| Total number of impact types categories considered in the method/ Total number of impact types categories | **21/41** | **13/41** | **13/41** | **8/41** | **21/41** | **18/41** | **9/41** | **32/41** | **12/41** | **19/41** | **23/41** | **14/41** | **17/41** | **29/41** | **6/41** |
| Total coverage (%) | **51** | **32** | **32** | **20** | **51** | **44** | **22** | **80** | **29** | **46** | **56** | **34** | **41** | **71** | **15** |
